# Secoiridoids from the Seed of *Gonocaryum calleryanum* and Their Inhibitory Potential on LPS-Induced Tumor Necrosis Factor and Nitric Oxide Production

**DOI:** 10.3390/molecules23071633

**Published:** 2018-07-04

**Authors:** Kun-Ching Cheng, Chi-I Chang, Yu-Chi Lin, Chih-I Liu, Yu-Ci Zeng, Yun-Sheng Lin

**Affiliations:** 1 Taiwan Sugar Research Institute, Tainan 70176, Taiwan; a64128@taisugar.com.tw or L38001048@mail.ncku.edu.tw; 2 Department of Chemistry, National Cheng Kung University, Tainan 701, Taiwan; 3 Department of Biological Science and Technology, National Pingtung University of Science and Technology, Pingtung 91201, Taiwan; changchii@mail.npust.edu.tw; 4 National Research Institute of Chinese Medicine, Taipei 11221, Taiwan; m8952612@hotmail.com; 5 Department of Nursing, Meiho University, Pingtung 91202, Taiwan; x00003077@meiho.edu.tw; 6 Department of Biological Science and Technology, Meiho University, Pingtung 91201, Taiwan; x00011052@meiho.edu.tw

**Keywords:** *Gonocaryum calleryanum*, secoiridoid, structure identification, anti-inflammatory

## Abstract

Three new secoiridoid constituents, goncarin A−C (**1**–**3**), and a new derivative, goncarin A monoacetate (**4**), along with two known lignins, pinoresinol (**5**) and paulownin (**6**), were isolated from the seed of *Gonocaryum calleryanum* (Baill.) Becc. The structures of the new metabolites were determined on the basis of extensive spectroscopic analysis, particularly mass spectroscopy and 2D NMR (^1^H–^1^H COSY, HMQC, HMBC, and NOESY) spectroscopy. The aim of this study was to identify the anti-inflammatory effects of compounds **1**–**6** on lipopolysaccharide (LPS)-stimulated murine macrophage cell lines (RAW 264.7). Following stimulation with LPS, elevated levels of nitric oxide (NO) production were detected in RAW 264.7 cells; however, pretreatment with compounds **1**–**6** significantly inhibited the production of NO (around 40–80%, *p* < 0.01–0.05), by suppressing the expression of inducible NO synthase (iNOS). In addition, LPS-stimulated tumor necrosis factor-α (TNF-α) production was significantly reduced by compounds **1**–**3** (25–40%, *p* < 0.01–0.05). These results suggested that compounds **1**–**3** may exert anti-inflammatory activity, and that compounds **1**–**3** may be considered a potential therapeutic for the treatment of inflammatory diseases associated with macrophage activation.

## 1. Introduction

The *Gonocaryum* Miq. plant is widely distributed in tropical and subtropical forests on coral rocks from Indonesia to the Philippines (Luzon and Batan Islands) and Taiwan (Hengchun Peninsula) [1]. *Gonocaryum calleryanum* (Baill.) Becc. is the only species of *Gonocaryum* found in tropical forests in southern Taiwan. Its leaves are used in Philippine traditional folk medicine for treating stomach disease [2]. However, there are only a few reports about the chemical composition of *Gonocaryum calleryanum*, and no reports on the analysis of biological activity and toxicity. Kaneko et al. [3] and Chan and co-workers [4] reported the isolation of secoiridoid glycosides, flavonoids, and flavonoid glycosides from the leaves, branch, stem, and root bark of this plant [5,6,7,8,9,10], but did not go any further to research its biological activity. The chemical composition of the seed has never been analyzed.

Inflammation is of importance in the highly complex immune response mounted to defend against harmful stimuli, including pathogens, damaged cells, or irritants. Macrophages, as critical participants in the inflammatory process, directly counteract the aforementioned stimuli [11]. The model most commonly used to investigate induced inflammation is the stimulation of macrophages by lipopolysaccharide (LPS) obtained from gram-negative bacteria [12]. The binding of LPS to its cognate receptors activates several signaling cascades that drive the expression of pro-inflammatory mediators and cytokines, including nitric oxide (NO) and tumor necrosis factor-α (TNF-α) [13]. 

NO is a highly reactive oxidant that is produced through the action of inducible NO synthase (iNOS), and participates in diverse biological mechanisms as a potent pro-inflammatory mediator [14]. Numerous studies have revealed that excessive NO production is important in the pathogenesis of inflammation and can lead to tissue damage by reacting with reactive oxygen species [15]. Therefore, suppressing the overproduction and activity of pro-inflammatory cytokines is necessary to reduce inflammation and its symptoms, and this method has proved to be successful in the treatment of certain inflammatory diseases, including rheumatoid arthritis, obesity, diabetes mellitus, cancer, and atherosclerosis [16]. In the present study, the anti-inflammatory effects of compounds **1**–**3** were evaluated using an LPS-stimulated RAW 264.7 macrophage cell model.

## 2. Results and Discussion

**Figure 1:**
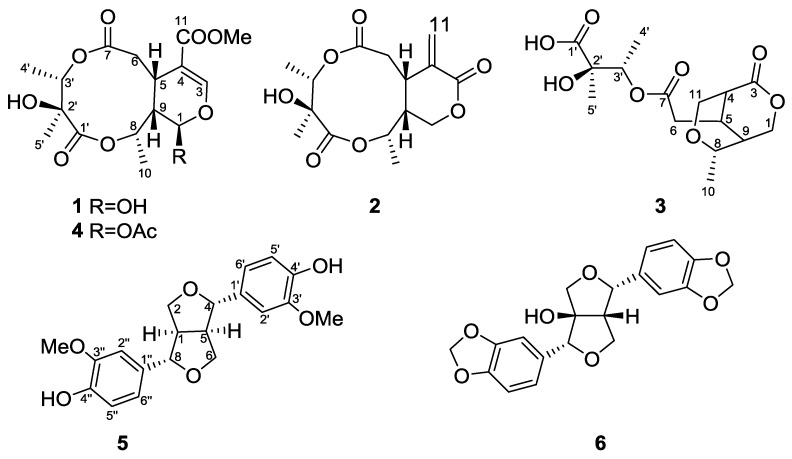
Chemical structures of compounds **1**–**6**.

The seeds of *G. calleryanum* were extracted with acetone, then suspended in H_2_O and extracted with EtOAc. The EtOAc-soluble part was subjected to extensive chromatography including Silica gel column, Sephadex LH-20 and reversed-phase HPLC, furnishing compounds **1**–**3** and **5**–**6** (Figure 1).

**Figure 2:**
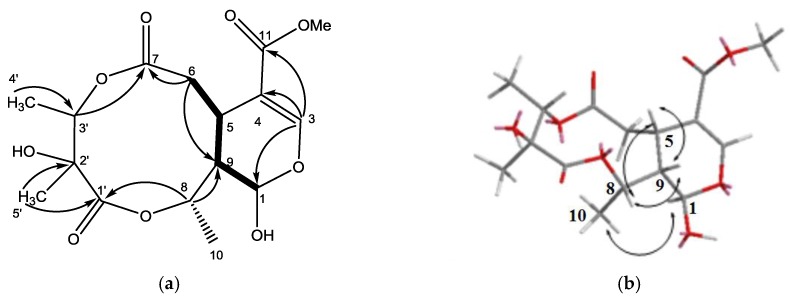
(**a**) The structure of metabolite **1** and selected ^1^H–^1^H COSY (▬) and HMBC (→) correlations. (**b**) Selective NOESY correlations of **1**.

The HR-ESI-MS of **1** (Supplementary Materials) revealed a pseudo-molecular-ion peak at *m*/*z* 381.1159 ([M + Na]^+^), consistent with the molecular formula C_16_H_22_O_9_, having six degrees of unsaturation. The IR spectrum displayed absorption bands diagnostic of OH (3456 cm^−1^), ester (1738 cm^−1^), and C=C bond (1635 cm^−1^) functionalities. The ^13^C-NMR spectrum of **1** showed the signals of a β-glucopyranosyl moiety, a trisubstituted double bond (δ_C_ 151.9 and 109.7), a carbomethoxyl group (δ_C_ 51.5 and 166.7), and a ketal carbon (δ_C_ 92.5). The presence of these partial structures suggested that **1** was an iridoid or secoiridoid. ^1^H- and ^13^C-NMR data (Table 1) indicated the presence of one OMe unit (δ_H_ 3.74 s, δ_C_ 51.5 q), two lactones, an ester group (δ_C_ 173.6 s, 175.7 s, 166.7 s), and one C=C bond (δ_H_ 7.52 s, δ_C_ 151.9 d, 109.7 s), accounting for 6 degrees of unsaturation and suggesting **2** additional rings. The ^1^H-NMR, ^13^C-NMR, and DPET spectrums also revealed one CH_2_ (δ_H_ 2.60 td, 3.10 td, δ_C_ 35.4 t), three CHO groups (δ_H_ 5.88 br s, δ_C_ 92.5 d; δ_H_ 4.94 q, δ_C_ 72.9 d; δ_H_ 5.05 q, δ_C_ 73.2 d), two CH (δ_H_ 3.36 m, δ_C_ 30.6 d; δ_H_ 1.98 br d, δ_C_ 43.9 d), three additional Me (δ_H_ 1.46 d, δ_C_ 18.3 q; δ_H_ 1.33 d, δ_C_ 12.8 q; δ_H_ 1.35 s, δ_C_ 16.4 q), and one additional quaternary C (δ_C_ 74.5 s). The COSY correlations showed the connection from H-1 to H-9, and H-5 to H-6 and H-9. The HMBC (Figure 2) showed correlations of H-3 to C-4, C-11, and C-1, H-3 to C-1 and C-5, and, with the aid of ^13^C-NMR spectrum, indicated that the COOMe group is attached to C-4 and the OH group is attached to C-1, assuming the position of the atom O is between C-1/C-3. The HMBC and ^13^C-NMR data also revealed the connections of H-6 to C-5, C-7, and C-9, H-3′ to C-7 and C-2′, Me-5′ to C-2′ and C-1′, H-8 to C-1′ and C-9, and indicated the position of two lactones and the OH groups. The relative configuration of **1** was determined on the basis of NOESY experiment and the references. The NOESY data exhibited the connectivity between H-8 to H-5 and H-9, and Me-10 to H-1. These findings and the references established the β-orientation of H-5, H-8, and H-9, and the α-configuration of H-1 and H-10. The stereochemical of C-2′ and C-3′ was determined by comparing the ^1^H and ^13^C-NMR spectra using alkaline hydrolysis of goncaryoside A [3,17]. It is speculated that if goncarin A undergoes alkaline hydrolysis, then it will obtain Kingiside aglycone and 2S, 3S angliceric acid [18,19,20,21,22]. Hence, the structure of goncarin A, one new natural compound, was established as **1**.

The HR-ESI-MS of **2** revealed a pseudo-molecular-ion peak at *m*/*z* 335.1105 ([M + Na]^+^), consistent with the molecular formula C_15_H_20_O_7_, having six degrees of unsaturation. The IR spectrum displayed absorption bands diagnostic of OH (3456 cm^−1^), ester (1734 cm^−1^), and C=C bond (1635 cm^−1^) functionalities. The ^1^H-NMR, ^13^C-NMR (Table 1), and DPET spectrum indicated the presence of three Me (δ_H_ 1.23 s, δ_H_ 1.31 d, 1.38 d), one C=CH_2_ (δ_H_ 5.68 s, δ_H_ 6.40 s; δ_C_ 128.8 t, 138.4 s), one CH_2_ (δ_H_ 2.33 m, 2.70 t, δ_C_ 36.0 t), one OCH_2_ group (δ_H_ 4.64 d, δ_C_ 64.5 t), two CH (δ_H_ 3.48 d, δ_C_ 40.4 d; δ_H_ 2.30 m, δ_C_ 41.6 d), two OCH groups (δ_H_ 4.82 q, δ_C_ 73.8 d; δ_H_ 5.06 q, δ_C_ 73.5 d), three lactones (δ_C_ 171.6 s, 174.9 s, 163.7 s), and one quaternary C (δ_C_ 74.3 s). Accounting for 6 degrees of unsaturation, **2** additional rings were suggested. The COSY correlations showed the connection of from H-1 to H-9, from H-9 to H-5, and from H-5 to H-6. The HMBC correlations of H-11 to C-4, C-5-, and C-3 exhibited that the C=C was at C-4 when the lactone was at C-3. The connections of the HMBC spectrum also showed the correlations of Me-4′ to C-3′, C-2′, and C-7, H-8 to C-9 and C-1′, and H-5′ to C-1′, C-2′, and C-3′, indicating the assignment of the lactone and the OH groups at C-7, C-1′, and C-2′, and connectivity of HMBC to complete the plane structure of **2**. The relative configuration of **2** was determined on the basis of NOESY experiment. The NOESY data exhibits the connectivity of H-8 to H-5 and H-9. These findings and the references established the β-orientation of H-5, H-8, and H-9, and the α-configuration of Me-10. The stereo chemical of C-2′ and C-3′ was determined by comparing the ^1^H and ^13^C-NMR spectra of goncarin A. Hence, the structure of the newly discovered natural compound, goncarin B, was established as **2**.

**Figure 3:**
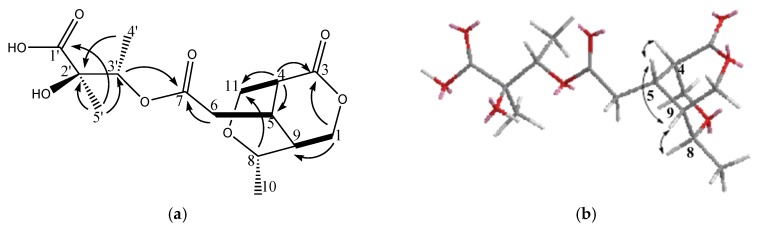
(**a**) The structure of metabolite **3** and selected ^1^H–^1^H COSY (▬) and HMBC (→) correlations. (**b**) Selective NOESY correlations of **3**.

The molecular formula C_15_H_22_O_8_ (five degrees of unsaturation) of **3** was deduced from the HR-ESI-MS data (*m*/*z* 353.1209 ([M + Na]^+^)). Its IR spectrum showed absorption bands suggesting the functionalities of OH (3433 cm^−1^) and ester (1738 cm^−1^). The ^1^H-NMR, ^13^C-NMR, and DEPT spectroscopic data (Table 1) indicated the presence of three CH_3_, two OCH_2_, two OCH, and three CH, and four quaternary C including one lactone, one ester group, and one acid group. Accounting for 5 degrees of unsaturation, **2** additional rings were suggested. In comparison with goncarin B, two sets of signals (δH 2.70 d, δH 3.75 m; δC 48.9 d, 61.1 t) were found from ^1^H-NMR and ^13^C-NMR. According to the COSY correlations, it showed the connection of H-1 to H-9, H-5 to H-6, and H-4 to H-11. The HMBC correlations of H-1 to C-3, C-5, and C-9, H-4 to C-3, C-5, and C-11, and H-6 to C-7, established lactones at C-3, the ester group at C-7, and the assignments of the right part of structure. The connections of HMBC spectrum also showed the correlations of H-3′ to C-7, C-2′, Me-4′, and Me-5′, Me-5′ to C-1′, C-2′, and C-3′, and Me-4′ to C-2′ and C-3′, which exhibited the location and the assignments of the left part of structure. From the above precise spectral data, it can be inferred that the oxygen-containing open ring at position C-8 of goncarin B is linked to the double bond on C-11. The NOESY spectrum showed the correlations of H-8 to H-5, H-4 to H-5, H-5 to H-9, and H-8 to H9. The relative configuration of **3**, elucidated mainly from the nuclear Overhauser effect spectroscopy (NOESY) spectrum, was compatible with that of **3** ascertained using molecular mechanics calculations (MM2). It is suggested to be the most stable conformations, as shown in Figure 3. These findings and the references established the β-orientation of H-4, H-5, H-8, and H-9, and the α-configuration of Me-10. Hence, the structure of newly discovered natural compound, goncarin C, was established as **3**.

## 3. Experimental Section

### 3.1. General

Prep. TLC: precoated silica-gel plates (SiO_2_; silica gel 60 F_254_, 1 mm; Merck KGaA, Frankfurter Strasse 250, Darmstadt, Germany). Column chromatography (CC): SiO_2_ 60 (Merck KGaA, Frankfurter Strasse 250, Darmstadt, Germany) or Sephadex LH-20 (GE Healthcare Bio-Sciences AB, Uppsala, Sweden). HPLC: Hitachi system; LiChrospher_ Si 60 (10 mm i.d. × 250 mm, 5 μm; Merck KGaA, Frankfurter Strasse 250, Darmstadt, Germany) for normal phase and LiChrospher® 100 RP-18 Endcapped (10 mm i.d. × 250 mm, 5 μm; Merck KGaA, Frankfurter Strasse 250, Darmstadt, Germany) for reversed-phase. Optical rotations: Jasco-DIP-1000polarimeter. IR and UV Spectra: Hitachi-T-2001 and Hitachi-U-3210 spectrophotometers, respectively. ^1^H- and ^13^C-NMR, COSY, HMQC, HMBC, and NOESY experiments: Bruker-FT-300 spectrometer; chemical shifts d in ppm rel. to Me4Si as an internal standard, coupling constants *J* in Hz. EI-MS and HR-ESI-MS: Jeol-JMS-HX-110 mass spectrometer; in *m*/*z* (rel. %).

### 3.2. Plant Material

The seeds of the plant *Gomocaryum calleryanum* were collected from tropical forests in southern Taiwan in July 2011. Plant identification and collection was conducted by Sheng-feng Hong of the Hengchun Research Center, Forestry Research Institute. Samples were collected and stored in the Specimen Room at Meiho University, Taiwan (Sample No.: 2011-07-2).

### 3.3. Extraction and Isolation

The fresh seeds were dried by cold drying and smashed, and then a 5.5 kg dry sample was collected. The dry sample was extracted with acetone (3 × 4 L) at room temperature, and then the acetone extract was concentrated. The dark-brown crude extract (520 g) was partitioned between EtOAc and H_2_O (1:1). The EtOAc layer (220 g) was subjected to CC (SiO_2_, *n*-hexane/EtOAc 20:1 ~ 1:1) and got 11 fractions. *Fr.* 11 (890.6 mg) was separated by reserved-phase HPLC (MeOH/H_2_O/CH_3_CN 60:35:5) and then further subjected to reversed-phase HPLC (MeOH/H_2_O/CH_3_CN 50:45:5): goncarin A (206.5 mg). *Fr*. 4 (98.5 mg), *Fr*. 6 (84.5 mg), and *Fr*. 7 (60.2 mg) were individually separated by CC (*Sephadex* LH-20, CH_2_Cl_2_/MeOH 1:1) and resulted in *Fr*. 4.3, *Fr*. 6.4, and *Fr*. 7.5. *Fr*. 4.3 was subject to reversed-phase HPLC (MeOH/H_2_O/CH_3_CN 60:35:5): Goncarin B (20.5 mg). *Fr*. 6.4 and *Fr.* 7.5 were separately subjected to reversed-phase HPLC (MeOH/H_2_O/CH_3_CN 50:45:5): goncarin C (18.8 mg), pinoresinol (3.0 mg) [23,24], and Paulownin (1.7 mg) [25].

### 3.4. Reaction

Acetylation of **1** (100 mg) was treated with acetic anhydride/pyridine (1:1) and left at room temperature for 24 h. Meanwhile, the work continued on the product with HPLC and resulted in the goncarin A monoacetate (**4**) 61 mg.

Goncarin A (**1**): colorless oil. [α]_D_ = +299.4 (*c* 0.3, CH_2_Cl_2_). IR (CH_2_Cl_2_): 3456 (OH), 1738 (ester), 1635 cm^−1^ (C=C). UV λ_max_ (MeOH): 242 nm. HR-ESI-MS: 381.1159 ([M + Na]^+^, C_16_H_22_NaO_9_^+^; calc. 381.1161).

Goncarin B (**2**): colorless oil. [α]_D_ = −51.1 (*c* 0.4, CH_2_Cl_2_). IR (CH_2_Cl_2_): 3456 (OH), 1734 (ester), 1635 cm^−1^ (C=C). UV λ_max_ (MeOH): 242 nm. HR-ESI-MS: 335.1105 ([M + Na]^+^, C_15_H_20_NaO_7_^+^; calc. 335.1107).

Gonocarin C (**3**): colorless oil. [α]_D_ = +100.5 (*c* 0.1, CH_2_Cl_2_). IR (CH_2_Cl_2_): 3433 (OH), 1738 (ester) cm^−1^. UV λ_max_ (MeOH): 227 nm. HR-ESI-MS: 353.1209 ([M + Na]^+^, C_15_H_22_NaO_8_^+^; calc. 353.1212).

Gonocarin A monoacetate (**4**): colorless oil. [α]_D_ = 319.0 (*c* 0.3, CH_2_Cl_2_). IR (CH_2_Cl_2_): 3456 (OH), 1738 (ester), 1635 cm^−1^ (C=C). UV λ_max_ (MeOH): 241 nm. HR-ESI-MS: 423.1264 ([M + Na]^+^, C_18_H_24_NaO_10_^+^; calc. 423.1267). ^1^H-NMR (CDCl_3_): δ 7.38 (1H, s, H-3), 6.67 (1H, d, *J* = 10 Hz, H-l), 5.02 (1H, q, *J* = 6.8 Hz, H-3′), 4.83 (1H, q, H-8), 3.70 (3H, s, OMe), 3.40 (1H, dt, 12.0, 6.8 Hz H-5), 2.38 (1H, m, H-6), 2.71 (1H, m, H-6), 2.16 (3H, s, OAc), 2.14 (1H, m, H-9), 1.28 (3H, s, 5′-Me), 1.30 (3H, d, *J* = 6.8 Hz, 4′-Me), 1.50 (3H, d, *J* = 6.8 Hz, 10-Me); ^13^C-NMR (CDCl_3_): δ20.3 (C-10), 12.7 (C-4′), 16.0 (C-5′), 33.2(C-5), 36.1(C-6), 43.7 (C-9), 73.3(C-3′), 75.2 (C-8), 74.3 (C-2′), 90.1 (C-l), 111.2 (C-4), 152.6 (C-3), 165.7 and 51.5 (COOMe), 171.3 (C-7), 175.3 (C-1′), 168.5 and 21.0(OAc).

### 3.5. Anti-Inflammatory Testing

#### 3.5.1. Materials

Lipopolysaccharide (LPS; Escherichia coli O111:B4) and dimethyl sulfoxide (DMSO) were purchased from Sigma-Aldrich (St. Louis, MO, USA). Fetal bovine serum (FBS), Dulbecco’s Modified Eagle’s Medium (DMEM), and phosphate buffer saline (PBS) were obtained from Gibco, Invitrogen (Carlsbad, CA, USA). The TNF-α ELISA kit were obtained from Mouse TNF-α ELISA (Max Deluxe Sets; BioLegend, San Diego, CA, USA). The MTS assay kit and NO assay kit were purchased from CellTiter 96 AQ Non-Radioactive Cell Proliferation Assay and Griess Reagent System (Promaga, Madison, WI, USA).

#### 3.5.2. Cell Culture and Treatment

RAW 264.7 mouse macrophage cells were obtained from the Bioresources Collection and Research Center (Hsinchu, Taiwan; BCRC No. 60001). The macrophages were cultured in Dulbecco’s Modified Eagle’s Medium (DMEM) supplemented with 10% heat-inactive fetal bovine serum (FBS) in a humidified atmosphere CO_2_ incubator (5% CO_2_ in air, ESCO, Singapore) at 37 °C. For the experiments, cells (1 × 10^5^) were seeded in a culture plate with 24 wells and maintained within the incubator. RAW 264.7 macrophages were pre-treated with LPS (Escherichia coli O111:B4) and culture medium mixture (100 ng/mL) for 6 h in a 37 °C incubator [26]. Subsequently, the isolated compounds (compounds **1**–**6**) were dissolved in dimethyl sulfoxide (DMSO) and added into LPS/medium mixture with final concentrations of 0.5, 1.0, and 2.0 µg/mL overnight. The positive and negative control were LPS/medium mixture and culture medium only.

#### 3.5.3. MTS Assay

RAW 264.7 macrophages were treated as described. After overnight incubation, the culture medium was removed, and then cells were washed with PBS. Two hundred µL of MTS reagent (Promaga, Madison, WI, USA) was added into each well for 1 h in a 37 °C incubator. The absorbance was measured using a plate reader (BioTek, Winooski, VT, USA) at a wavelength of 490 nm [27].

#### 3.5.4. Enzyme-Linked Immunosorbent Assay Analysis

RAW 264.7 were cultured in a 24-well plate with LPS/medium mixture for 6 h, and then incubated with 3 different concentrations (0.5, 1.0, and 2.0 µg/mL) of compounds **1**–**6** overnight. Supernatants were collected and measured using a mouse TNF-α ELASA kit (Mouse TNF-α ELISA Kit KMC 3012; Invitrogen, Carlsbad, CA, USA) following manufacturer’s protocol [28].

#### 3.5.5. Nitric Oxide Assay

RAW 246.7 cells (1 × 10^5^) were seeded in a culture plate with 24 wells and maintained within the incubator. RAW 264.7 macrophages were pre-treated with LPS (Escherichia coli O111:B4) and culture medium mixture (100 ng/mL) for 6 h in a 37 °C incubator. Subsequently, compounds **1**–**6** were dissolved in dimethyl sulfoxide (DMSO) and added into LPS/medium mixture with final concentrations of 0.5, 1.0, and 2.0 µg/mL overnight. The amount of NO in the supernatants was detected by using the Griess Reagent System (Promaga, Madison, WI, USA) according to the manufacturer’s protocol. Data calculations were performed using MS-Excel 2010 software [29].

The pro-inflammatory cytokine TNF-α and the reactive free radical NO synthesized by inducible nitric oxide synthase (iNOS) are important macrophage-derived inflammatory mediators. Thus, the effect on the excessive productions of TNF-α and NO can be employed as criteria to evaluate the anti-inflammatory activity of test compounds. In this study, two inflammatory parameters (NO and TNF-α) were evaluated using macrophages incubated with and without LPS (basal values) and in the absence or presence of different concentrations of compounds **1**–**6** (0.5, 1.0, and 2.0 μg/mL).

**Figure 4:**
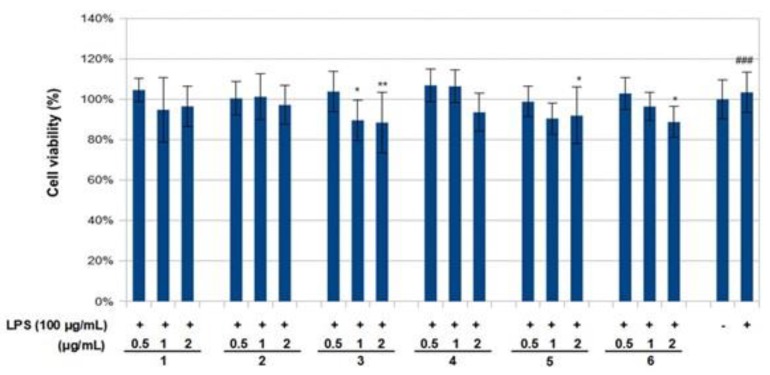
Effects of compounds **1**–**6** and lipopolysaccharide (LPS) on the viability of RAW 264.7 macrophages. Cells were treated with LPS for 6 h prior to treatment with indicated concentrations of compounds or LPS alone. Following a 24 h incubation period, cell viability was assessed using an MTS assay. Cell viability was calculated as the percentage of surviving cells over control cells (no compounds added). Values are presented as mean ± standard deviation of three independent experiments. ^###^ *p* < 0.001 control group as compared to LPS-treated group. * *p* < 0.05 and ** *p* < 0.01 were compared with the LPS-alone group. -: cells without treatment, +: cells previously treated with LPS.

**Figure 5:**
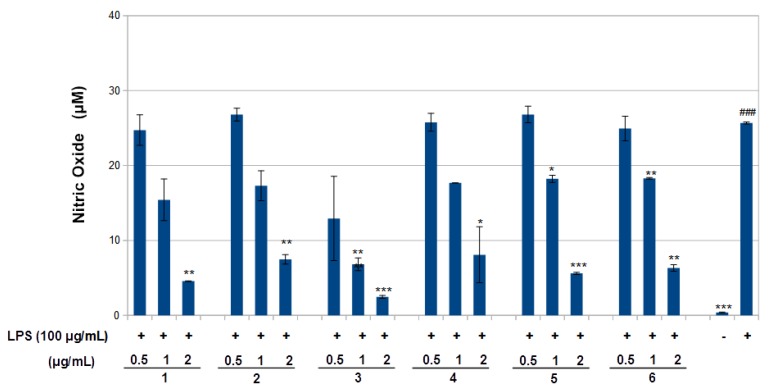
Inhibition of nitric oxide (NO) production by compounds **1**–**6** in LPS-stimulated RAW 264.7 macrophages. RAW 264.7 cells were stimulated by LPS (100 ng/mL) for 6 h and then treated with the indicated concentrations of compounds 1–6 for 24 h. NO was measured using Griess reagent. Values are presented as mean ± standard deviation of three independent experiments. ^###^ *p* < 0.001 control group as compared to LPS-treated group. * *p* < 0.05, ** *p* < 0.01, and *** *p* < 0.001 were compared with the LPS-alone group. -: cells without treatment, +: cells previously treated with LPS.

**Figure 6:**
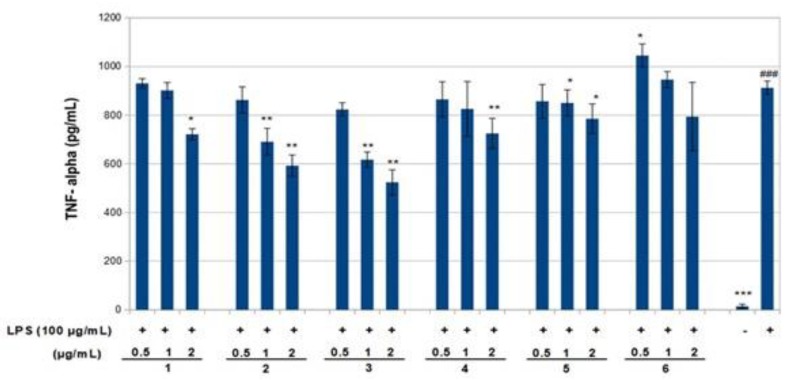
Inhibition of tumor necrosis factor-α (TNF-α) production by compounds **1**–**6** in LPS-stimulated RAW 264.7 macrophages. RAW 264.7 cells were stimulated by LPS (100 ng/mL) for 6 h then treated with various concentrations of compounds **1**–**6** for 24 h. TNF-α production was measured using the corresponding ELISA kits. Values are presented as mean ± standard deviation of three independent experiments. ^###^ *p* < 0.001 control group as compared to LPS-treated group. * *p* < 0.05, ** *p* < 0.01, and *** *p* < 0.001 were compared with the LPS-alone group. -: cells without treatment, +: cells previously treated with LPS.

The effect of compounds **1**–**6** on RAW 264.7 cell viability was determined by an MTS assay. Cells cultured with compounds **1**–**6** at concentrations of 0.5, 1.0 and 2 μg/mL used in the presence of 100 ng/mL LPS for 24 h did not change cell viability (Figure 4). The results obtained in the NO assay are shown in Figure 5. LPS significantly increased NO with respect to basal cells. Compound **1**–**6** significantly reduced the effect of LPS stimulation on nitric oxide positively according to concentration (around 40–80%, *p* < 0.01–0.05). The results obtained for the TNF-α assay are shown in Figure 6. LPS increased the TNF-α basal level significantly. Compounds **1**–**3** decreased LPS-stimulated TNF-α positively related to concentration (around 25–40%, *p* < 0.01–0.05), in comparison with the group of cells treated with LPS but without treatment with this compound. Compounds **1**–**3** exerted an effect not only on NO but also on TNF-α levels in a model of murine macrophages activated with LPS. With increasing concentration of compound added, both NO and TNF-α decreased, suggesting an anti-inflammatory action.

## 4. Conclusions

The inhibitory effects of compounds **1**–**6** on LPS-induced nitric oxide (NO) production and LPS-induced tumor necrosis factor-α (TNF-α) expression were determined by measuring the levels of nitrite and cytokine enzyme-linked immunosorbent assays (ELISAs). It was identified that compounds **1**–**3** may suppress NO production in LPS-activated RAW 246.7 macrophages by inhibiting inducible NO synthase (iNOS) expression, and exert curative effects on anti-inflammatory activity by reducing TNF-α expression. The results provide evidence in favor of the use of compounds **1**–**3** as a potential therapeutic for the treatment of inflammatory diseases.

## Figures and Tables

**Table 1:** ^1^H-NMR and ^13^C-NMR data for compounds **1**−**3**^a^.

	1		2		3	
No.	δ_H_	δ_C_	δ_H_	δ_C_	δ_H_	δ_C_
1	5.88 brs	92.5	4.62 (d,12.0)	64.5	4.61 (d,11.6)	65.1
3	7.52 s	151.9		163.7		172.3
4		109.7		138.4	2.70 m	48.9
5	3.36 m	30.6	3.48 (d,12.4)	40.4	2.88 m	35.9
6α	3.10 (dd, 15.2, 2.8)	35.4	2.70 (t, 15.2)	36	2.32 m	30.5
6β	2.60 (dd, 15.2, 2.8)		2.33 m		2.50 m	
7		173.6		171.6		171.8
8	4.94 (q, 4.8)	72.9	4.82 (q, 6.8)	73.8	4.81 (d, 6.4)	74.2
9	1.98 (brd, 1.6)	43.9	2.30 m	41.6	2.32 m	43.1
10	1.46 (d, 6.8)	18.3	1.38 (d, 6.8)	18.6	1.38 (d, 6.4)	18.7
11		166.7	5.68 s	128.8	3.75 m	61.1
			6.40 s		3.92 m	
OMe	3.74 s	51.5				
1′		175.7		174.9		174.8
2′		74.5		74.3		74.2
3′	5.05 (q, 6.4)	73.2	5.06 (q, 6.8)	73.5	5.05 (q, 6.4)	74.1
4′	1.33 (d, 6.4)	12.8	1.31 (q, 6.8)	12.7	1.35 m	12.7
5′	1.35 s	16.4	1.23 s	16.7	1.31 m	16.7

^a^ Assignments aided by the HMQC, HMBC, and NOESY experiments; ^1^H and ^13^C-NMR were measured at 400 and 100 MHz in CDCl_3_.

## Supplementary Materials

The physical and spectroscopic data of compounds **1**–**6**, as well as NMR and MS spectra of compounds **1**–**6,** are available online.
