# Supplementary material for: Secoiridoids from the Seed of Gonocaryum calleryanum and Their Inhibitory Potential on LPS-Induced Tumor Necrosis Factor and Nitric Oxide Production
Source: Molecules. 2018 Jul 4;23(7):1633. doi: 10.3390/molecules23071633 (PMC6100119; doi:10.3390/molecules23071633)
Supplement: Supplementary file 1 [file molecules-23-01633-s001.pdf]

¹H NMR spectrum of Gonocarin A （1）

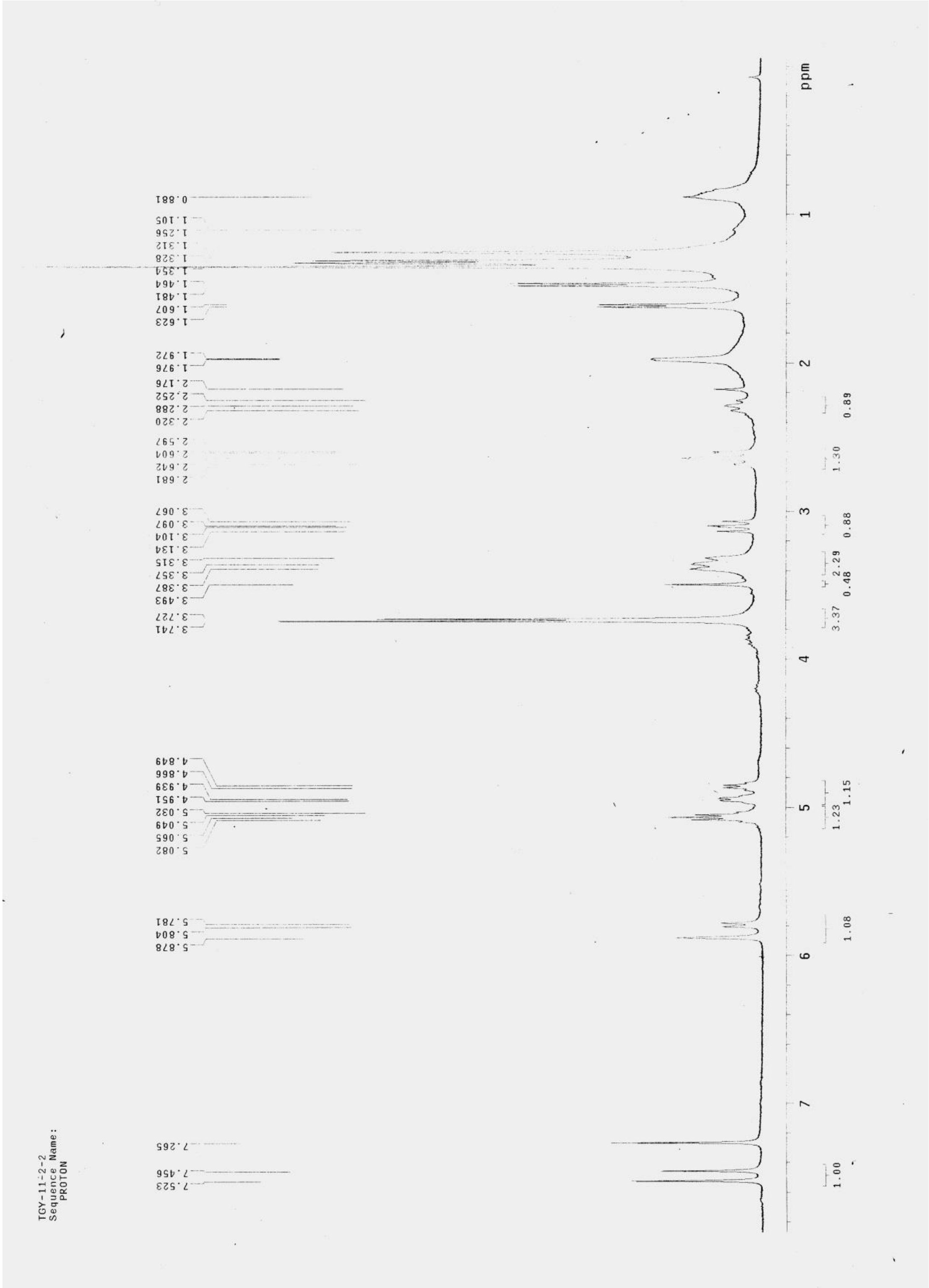

^{13}C NMR spectrum of Gonocarin A （1）

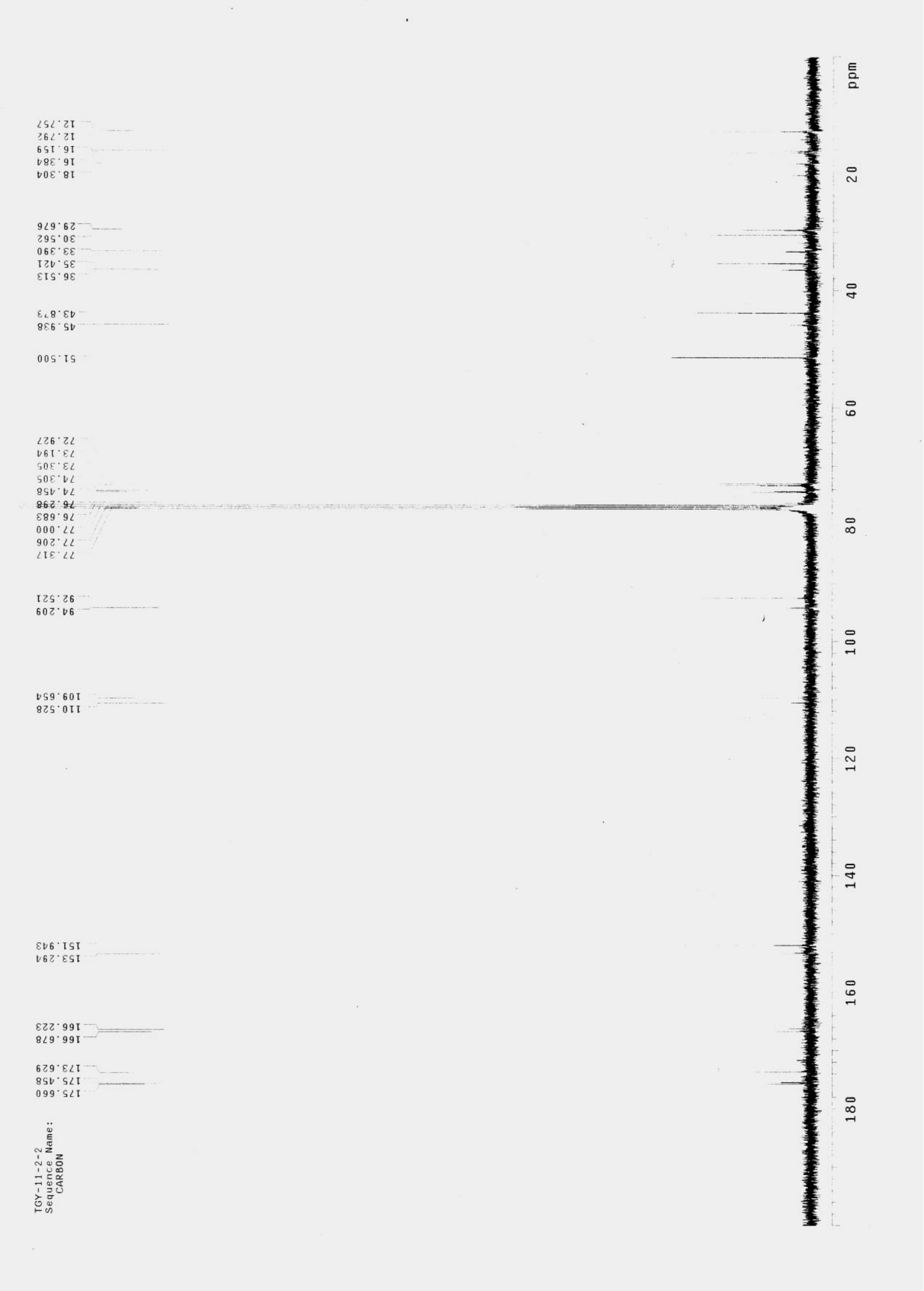

COSY spectrum of Gonocarin A （1）

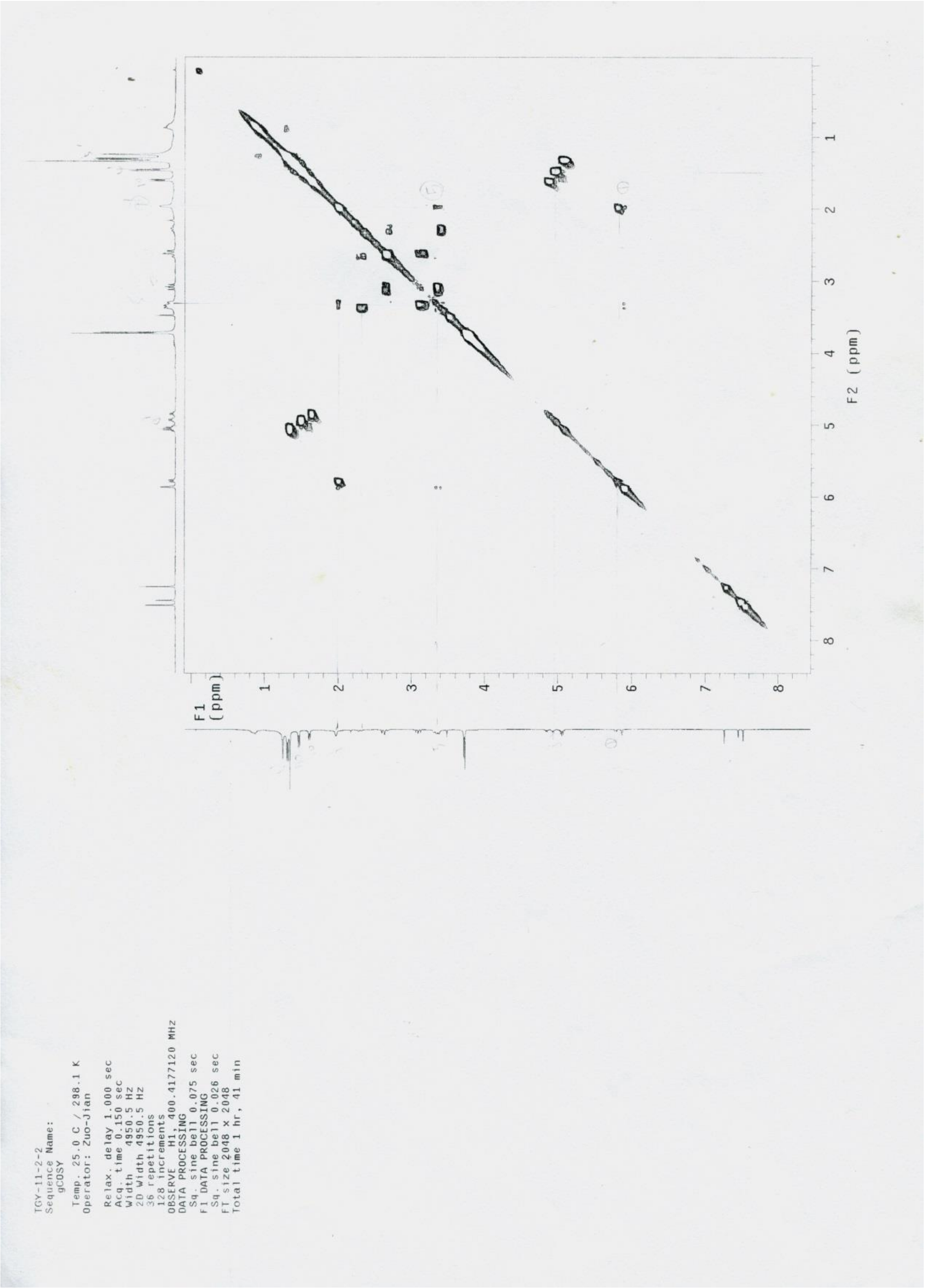

HMQC spectrum of Gonocarin A （1）

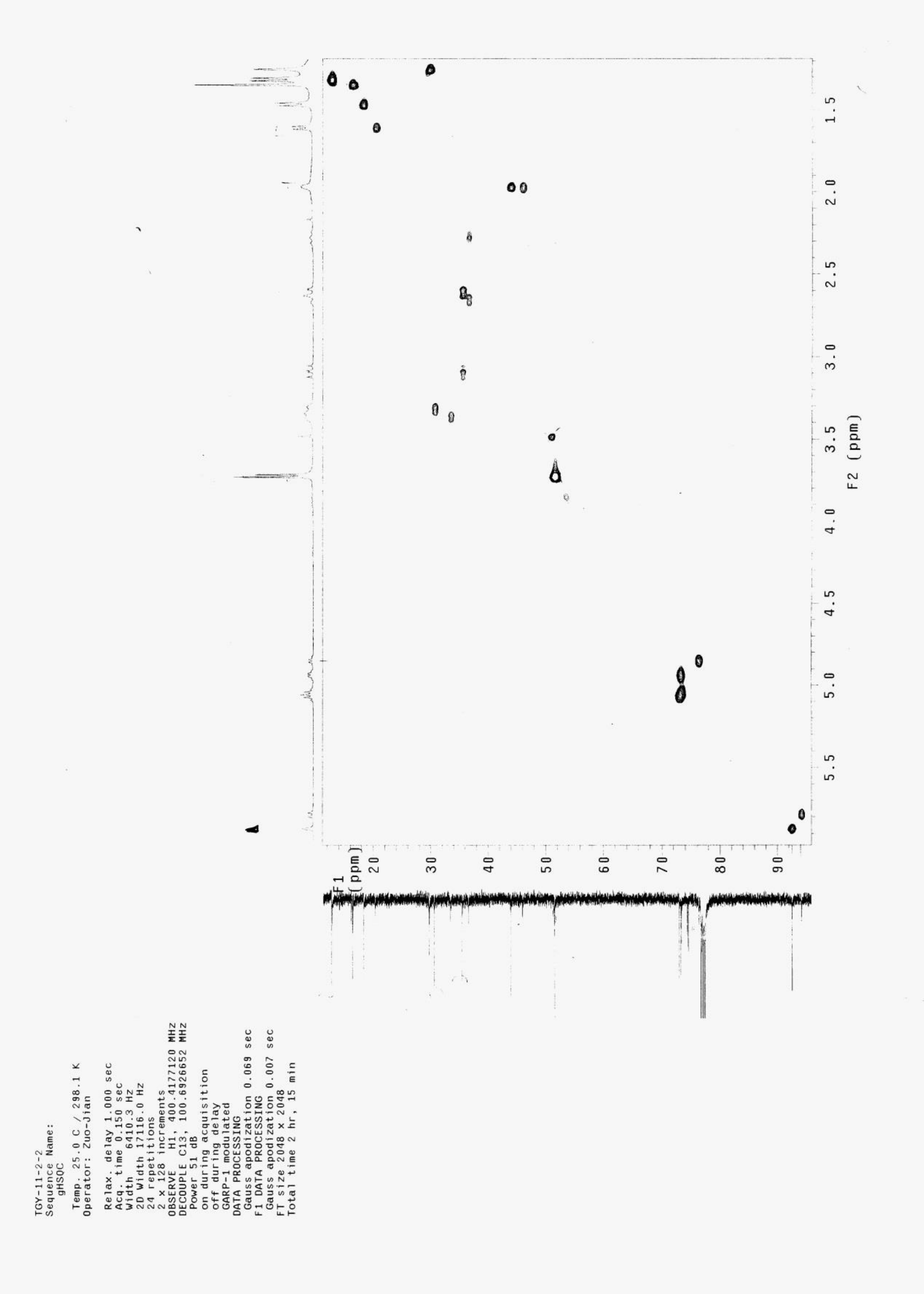

HMBC spectrum of Gonocarin A （1）

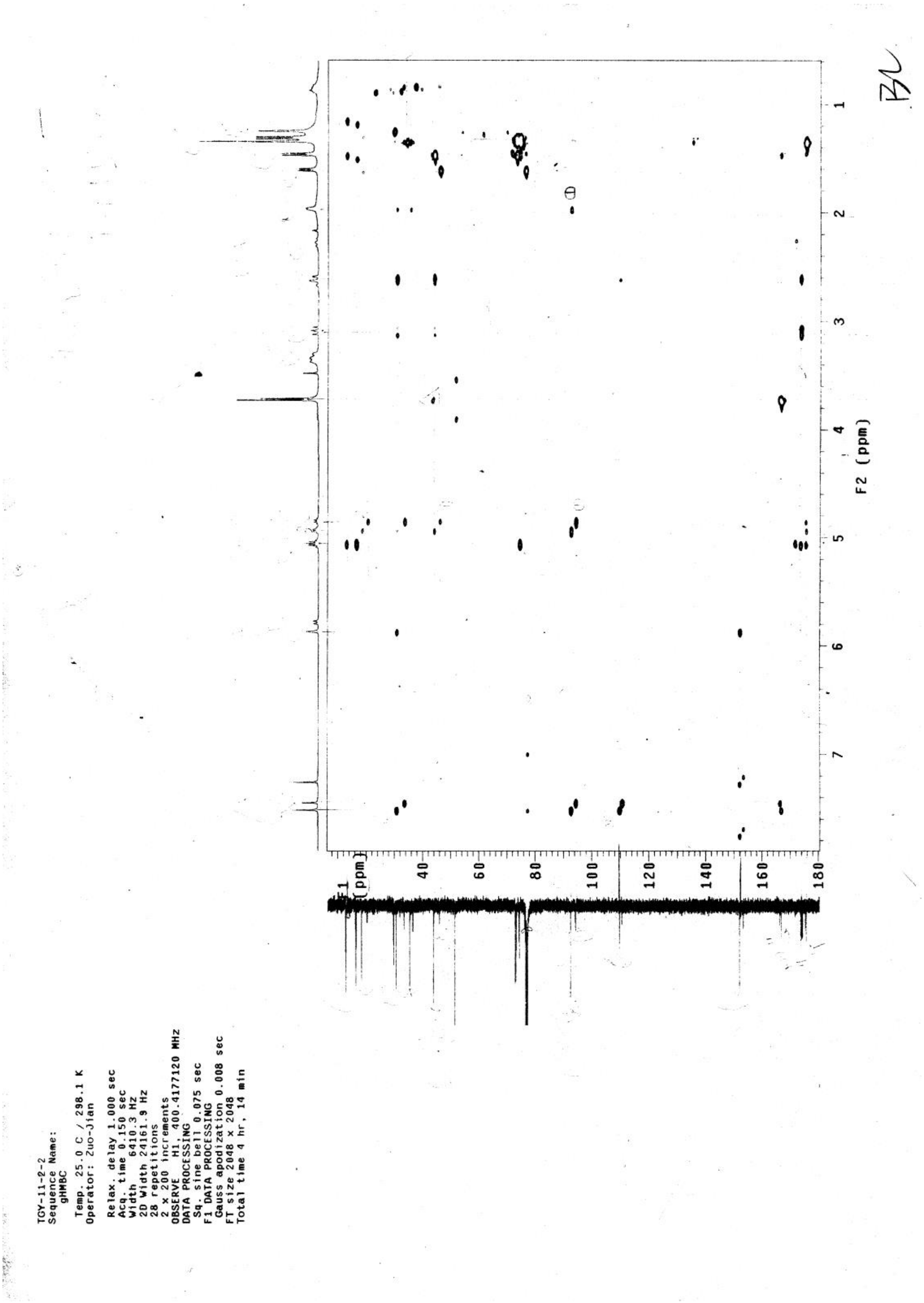

NOESY spectrum of Gonocarin A （1）

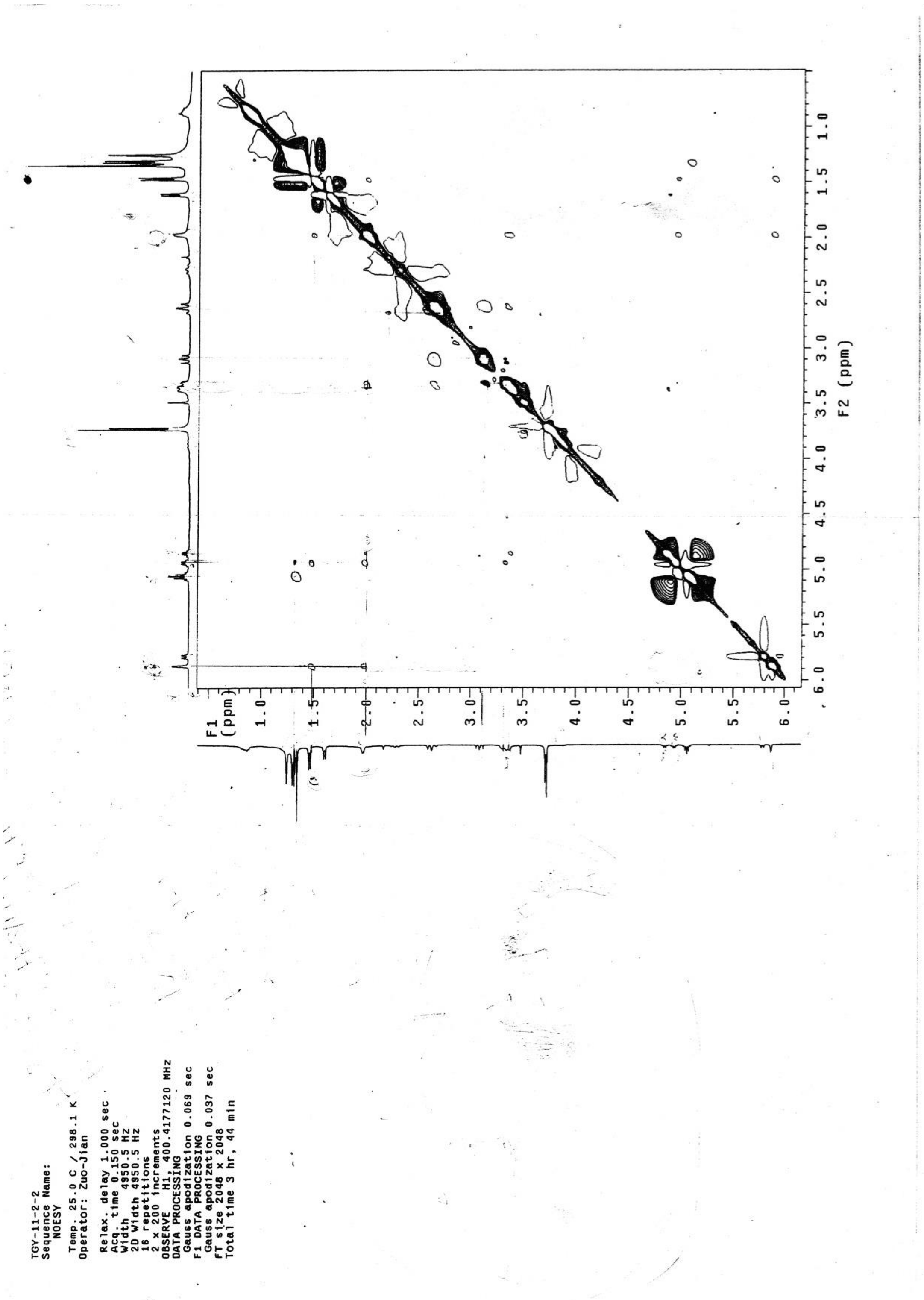

IR spectrum of Gonocarin A （1）

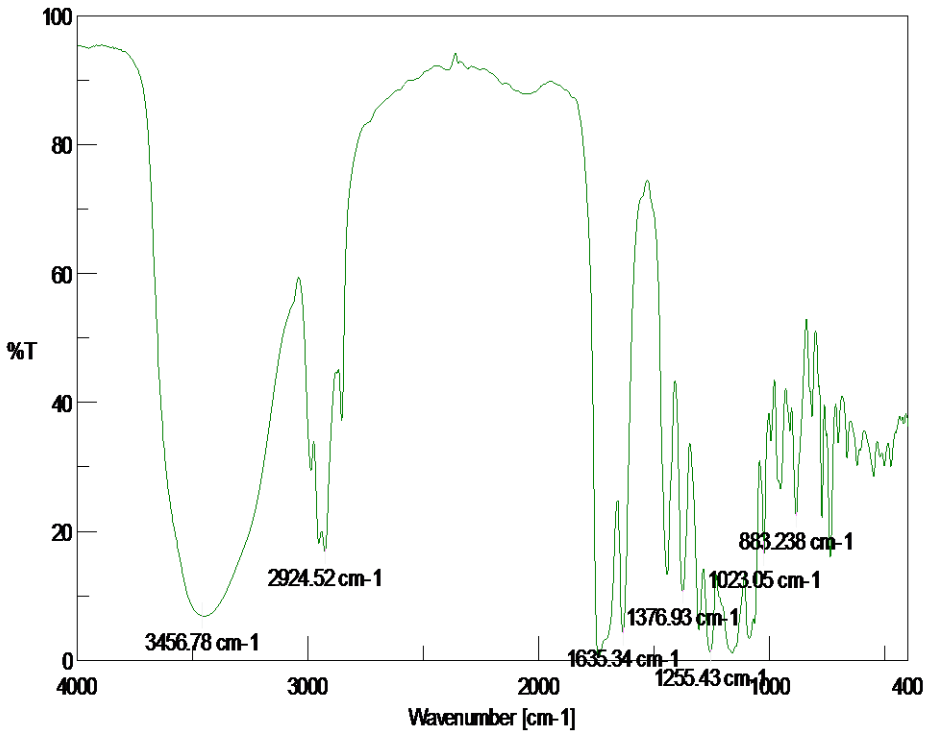

HRESIMS spectrum of Gonocarin A （1）

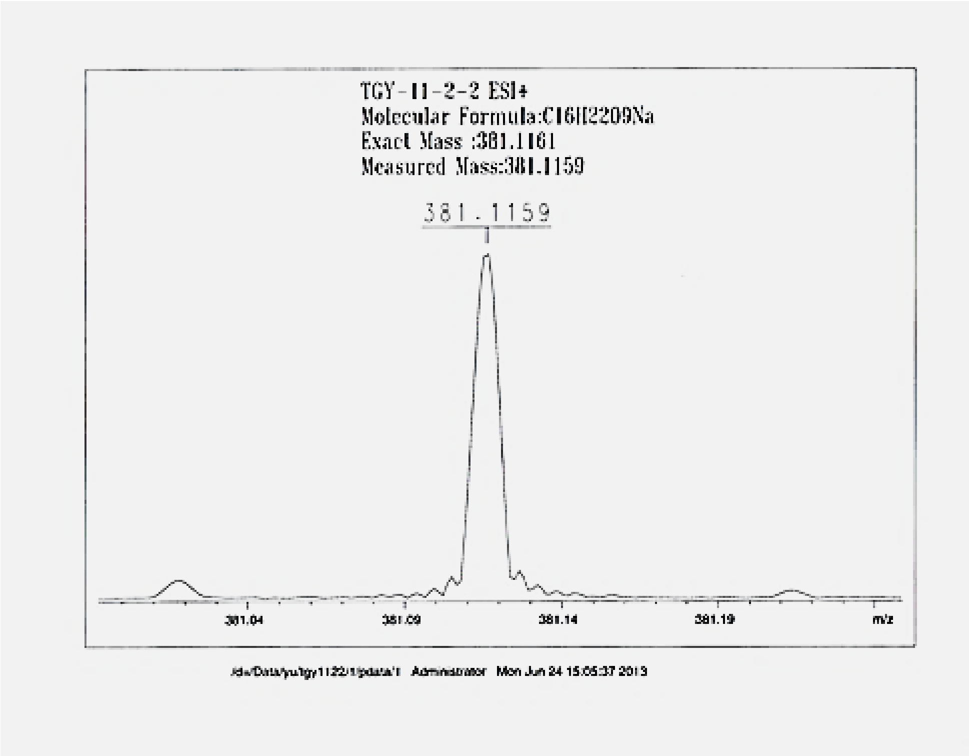

^{1}H NMR spectrum of Gonocarin B （2）

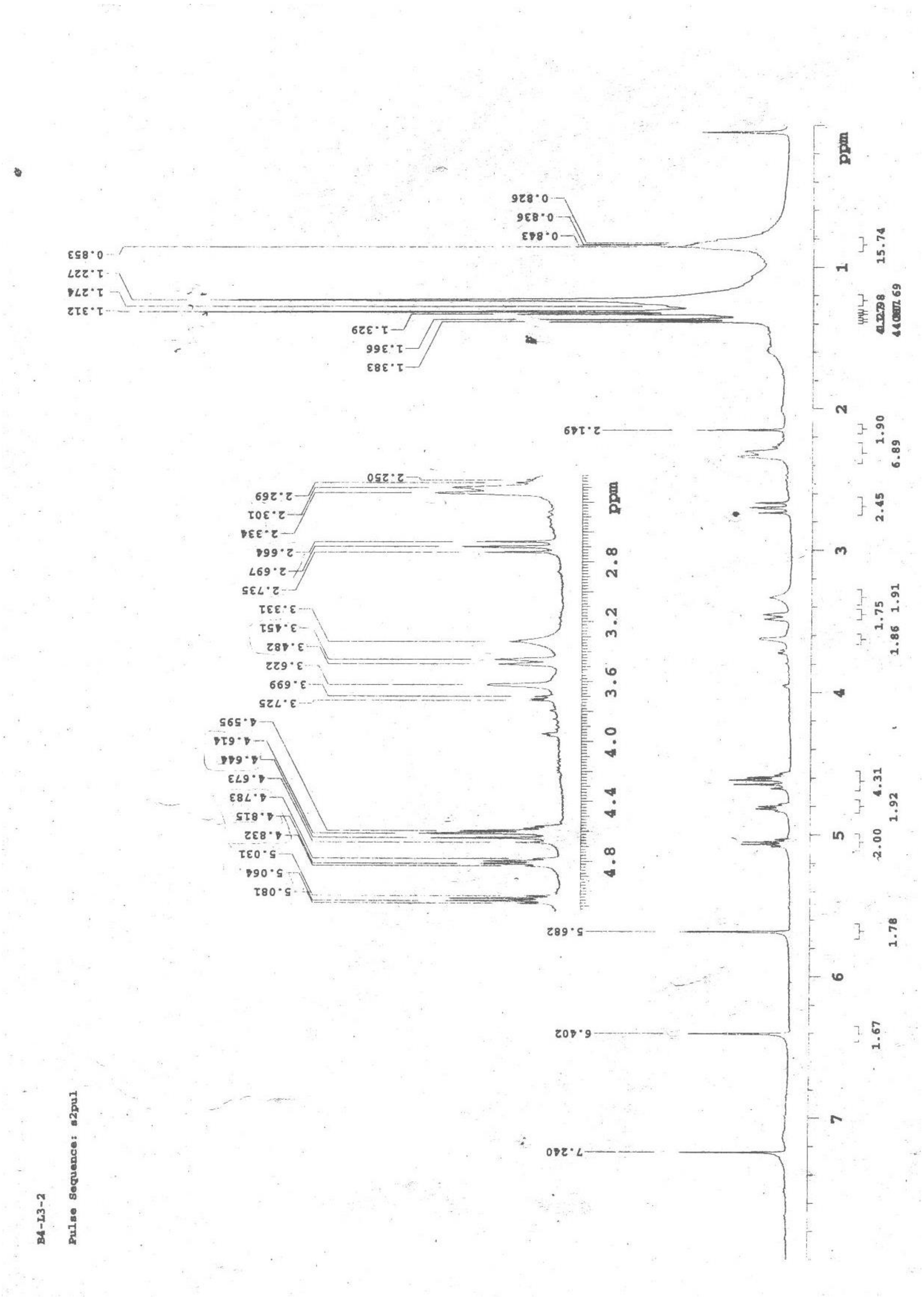

^{13}C NMR and DEPT spectrum of Gonocarin B （2）

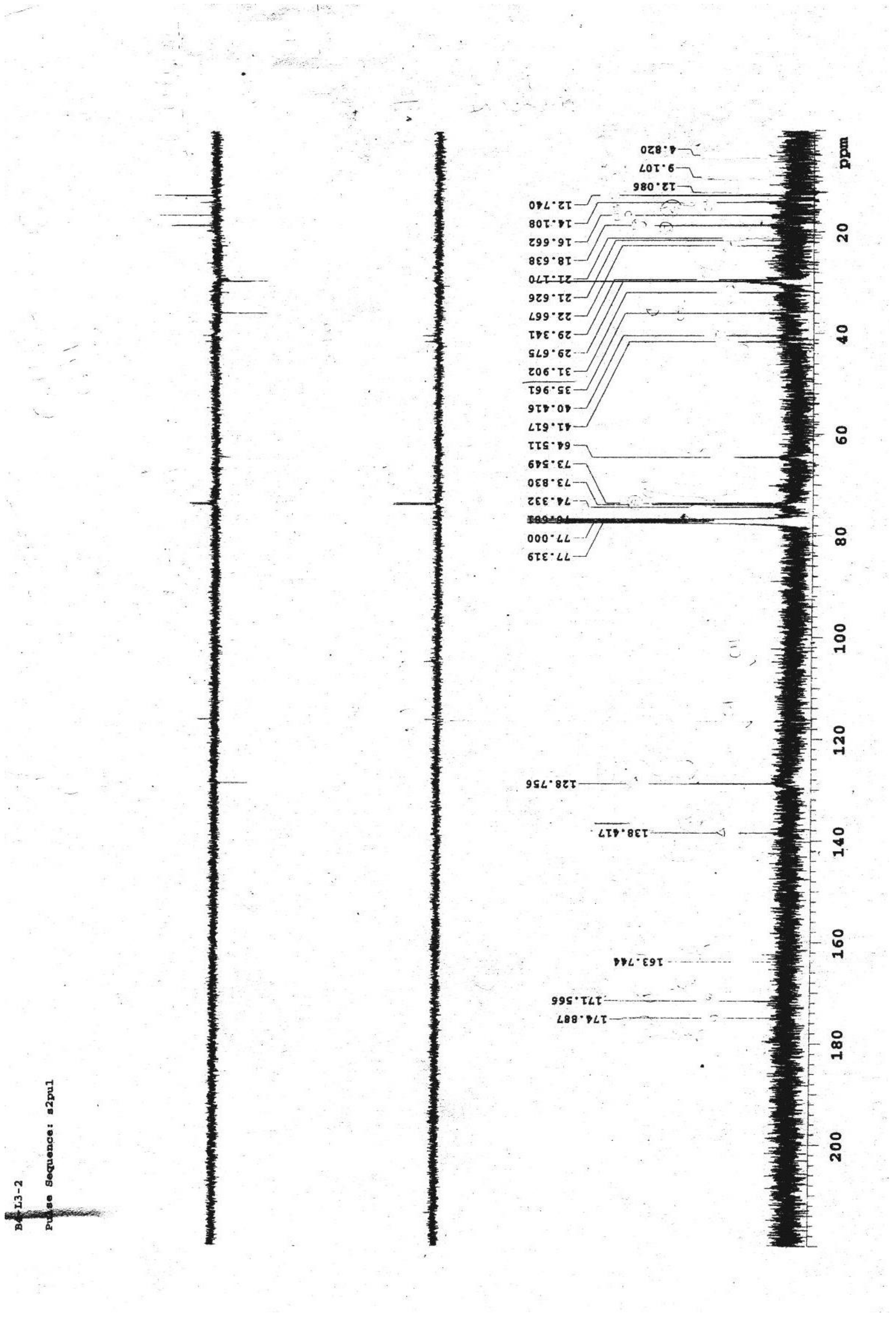

COSY spectrum of   Gonocarin B （2）

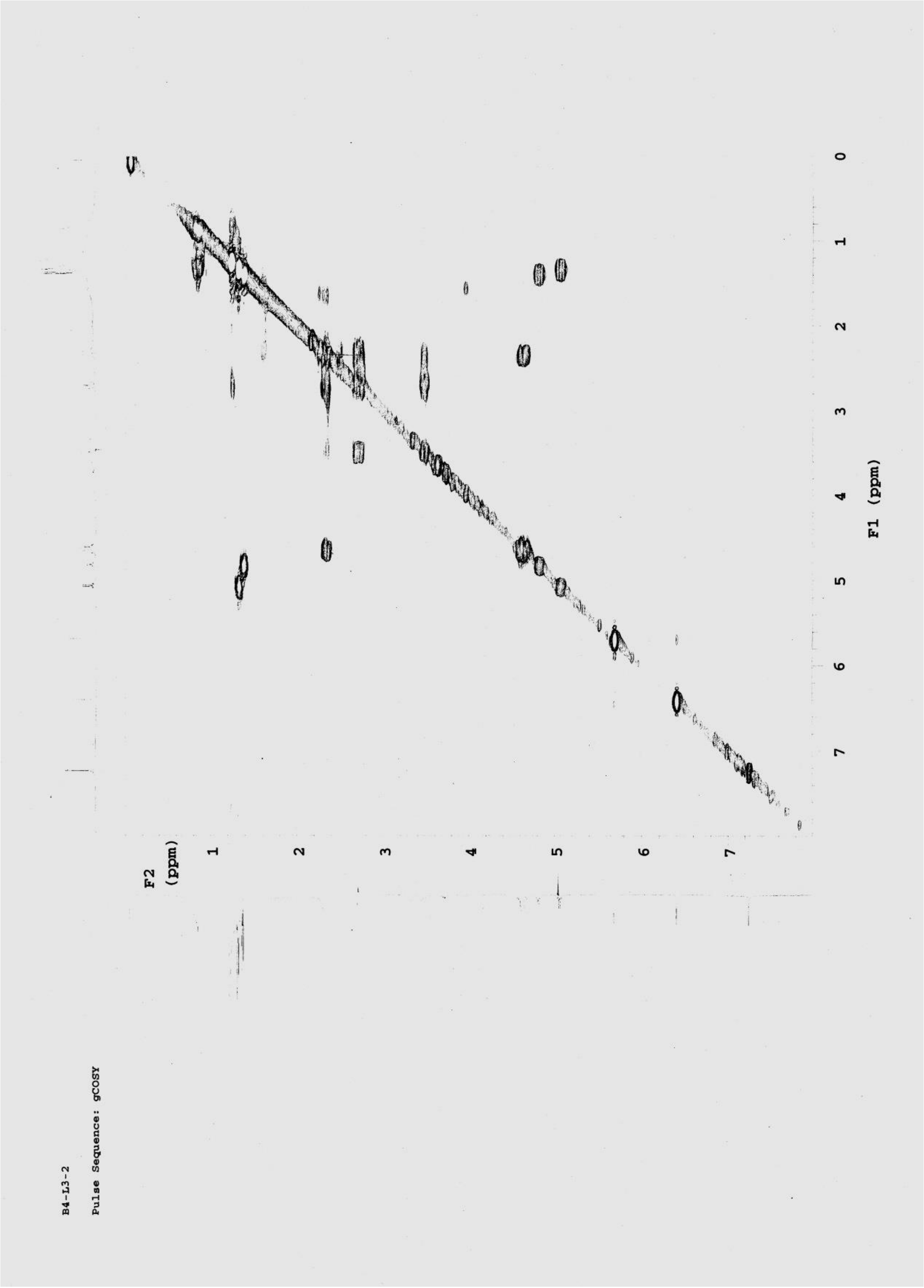

HMQC spectrum of Gonocarin B （2）

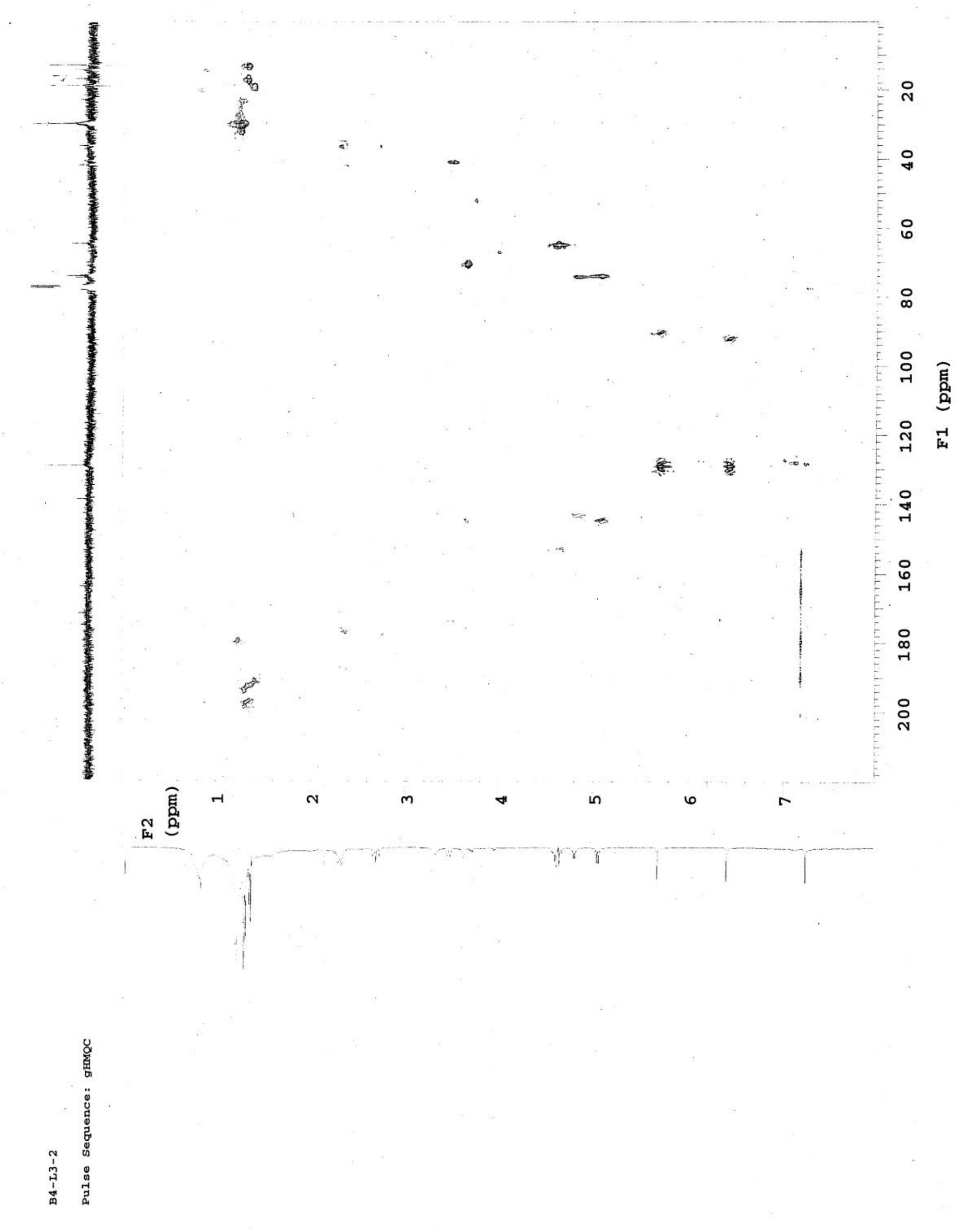

HMBC spectrum of Gonocarin B （2）

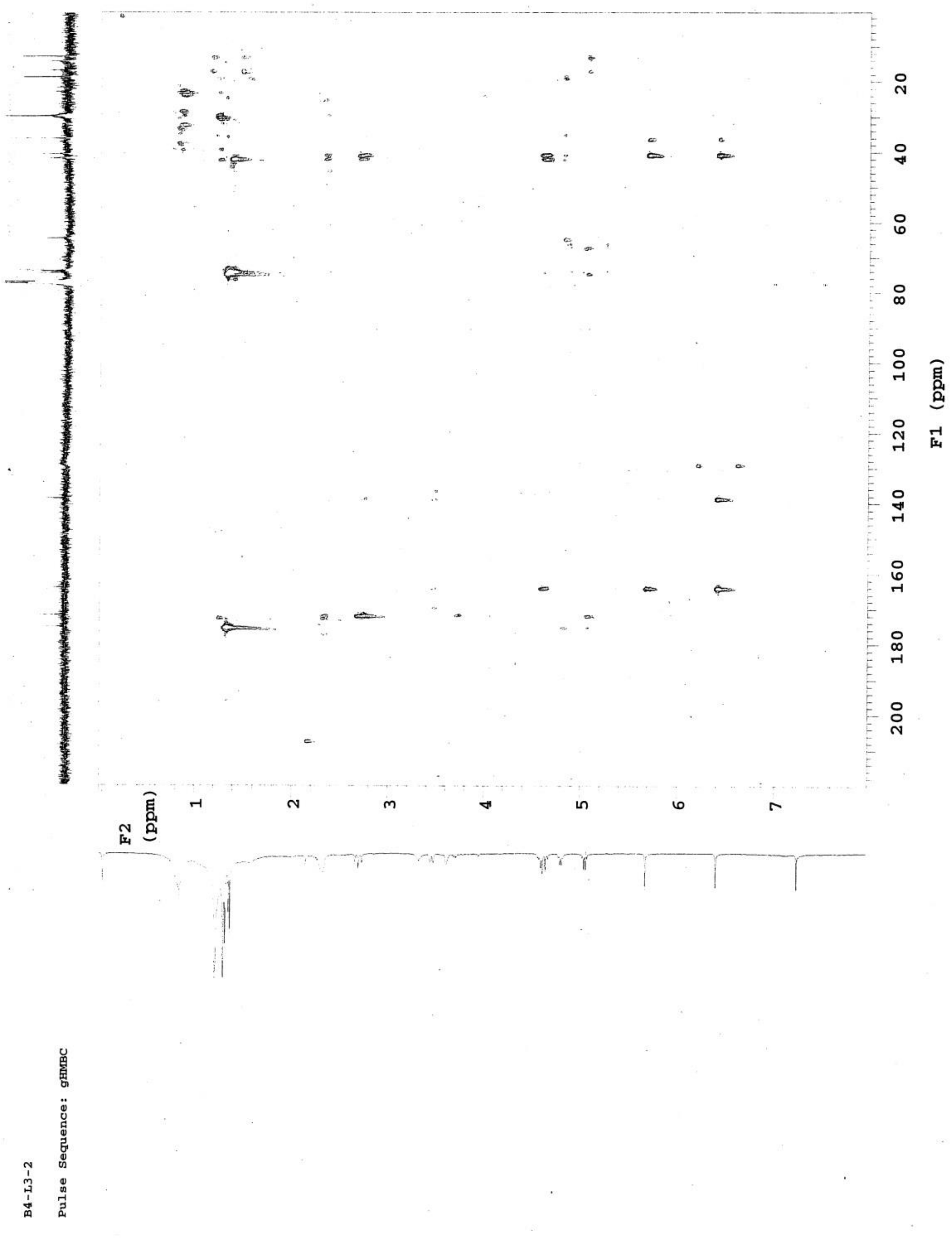

NOESY spectrum of Gonocarin B （2）

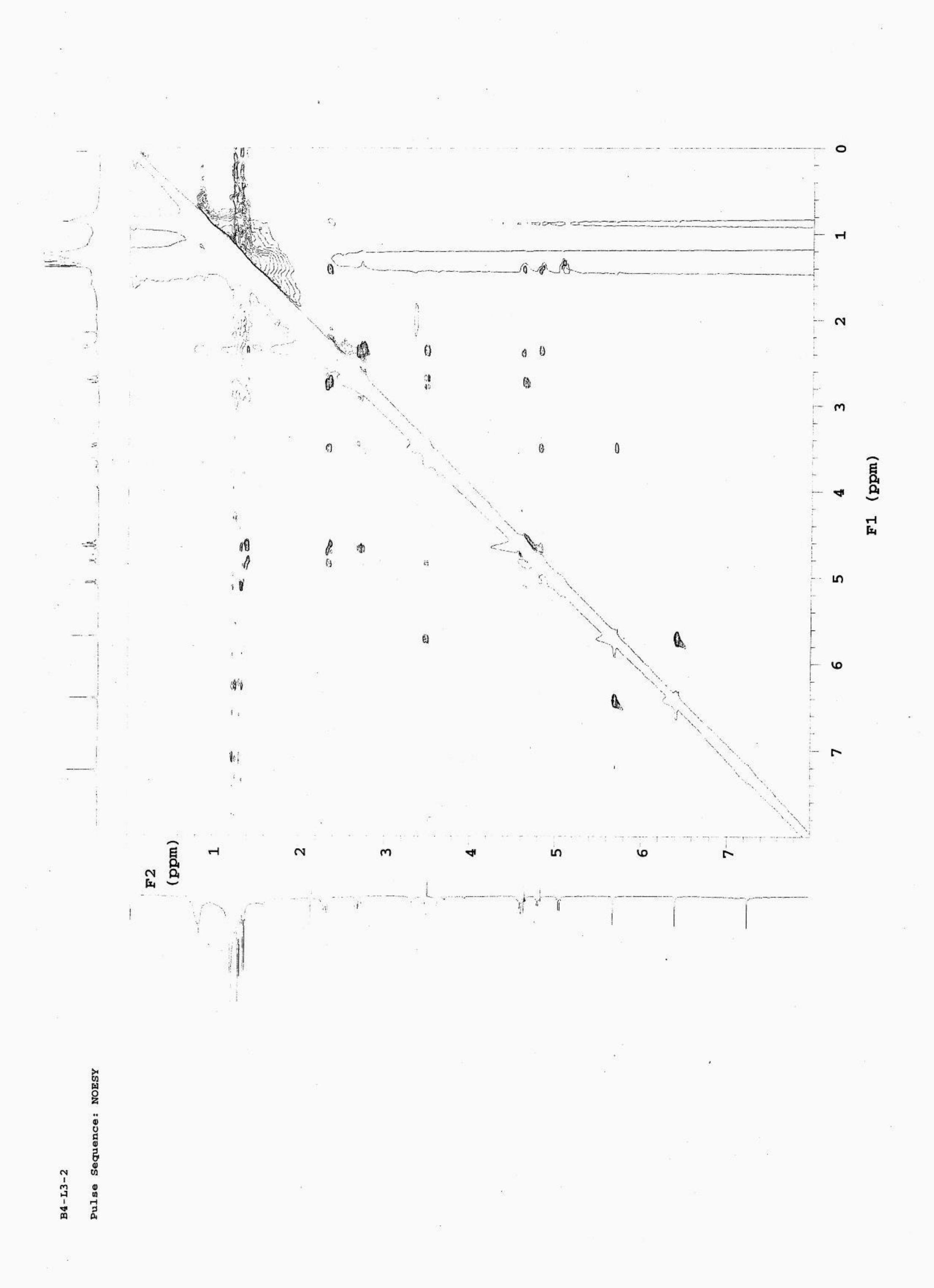

IR spectrum of Gonocarin B （2）

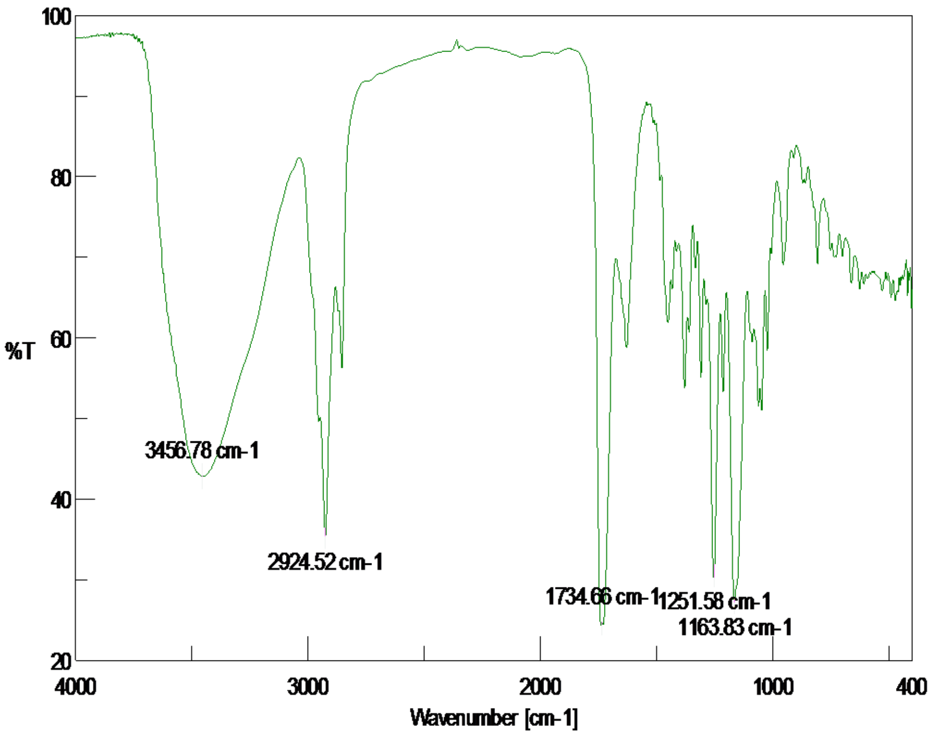

HR-ESI-MS spectrum of Gonocarin B （2）

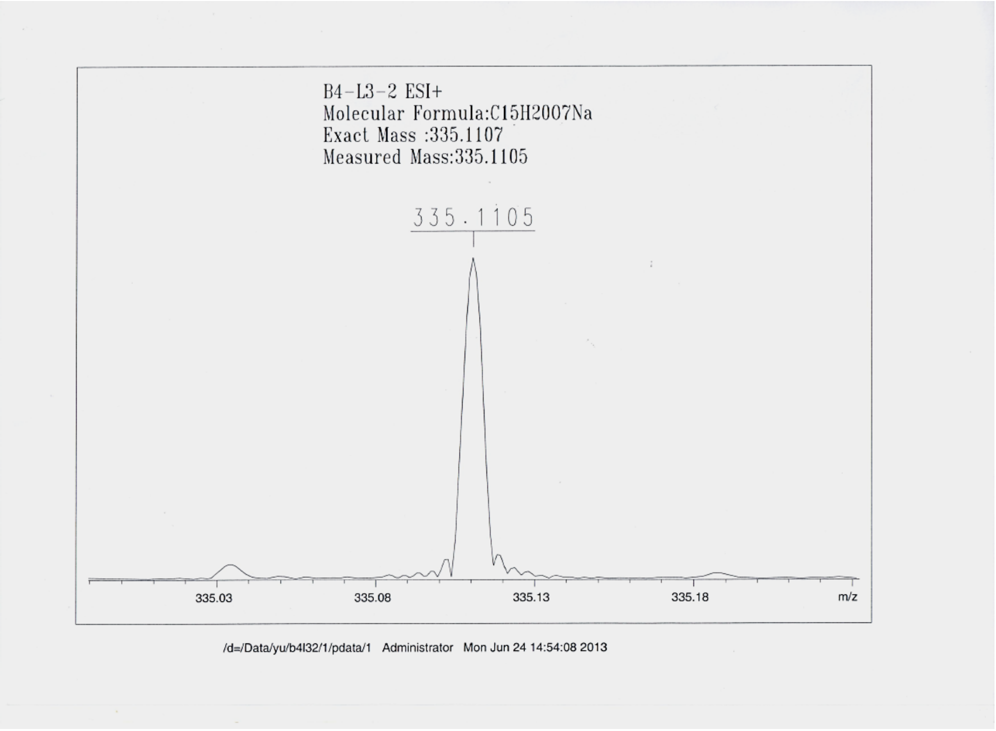

^{1}H NMR spectrum of Gonocarin C （3）

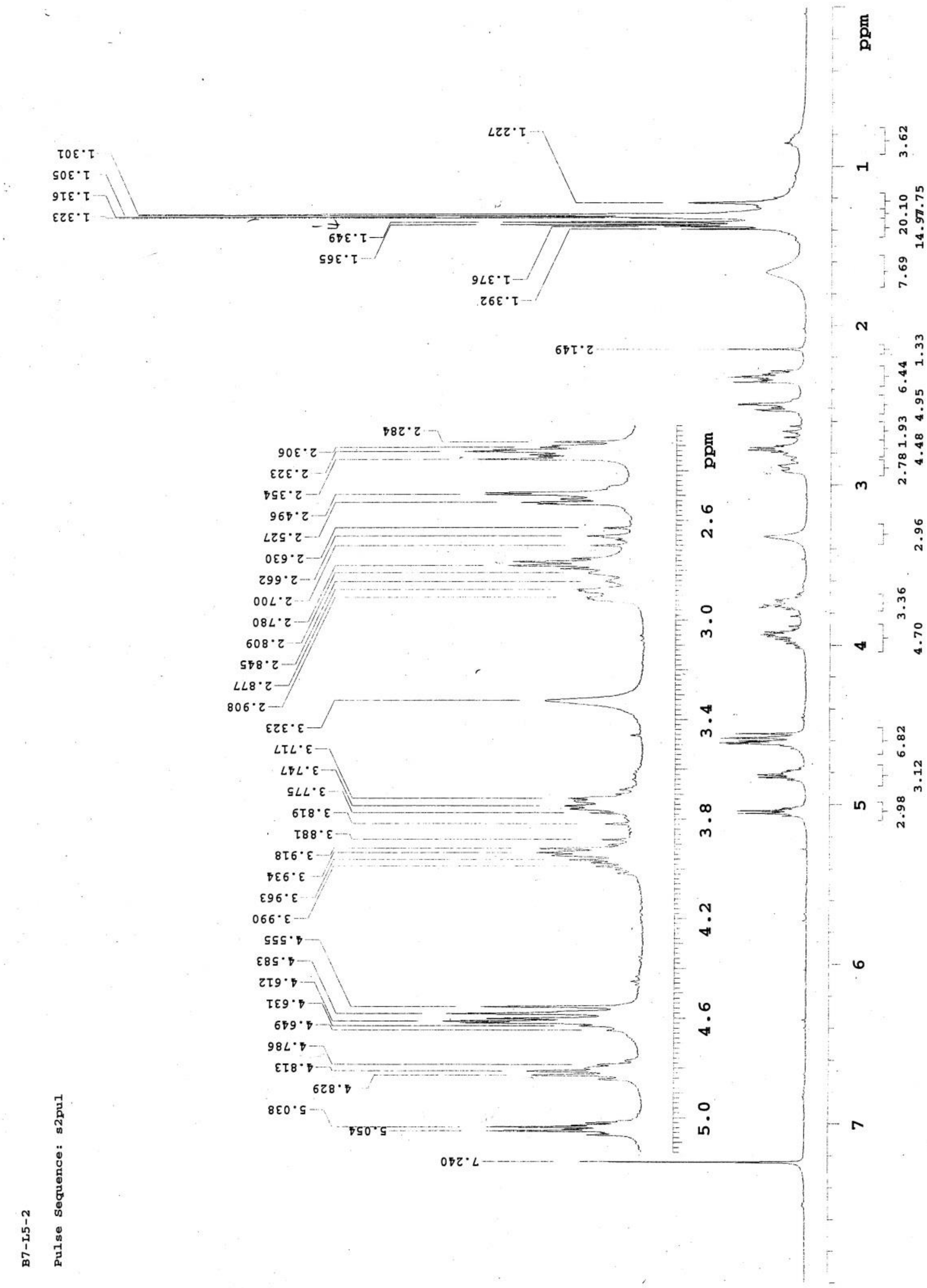

^{13}C NMR spectrum of Gonocarin C （3）

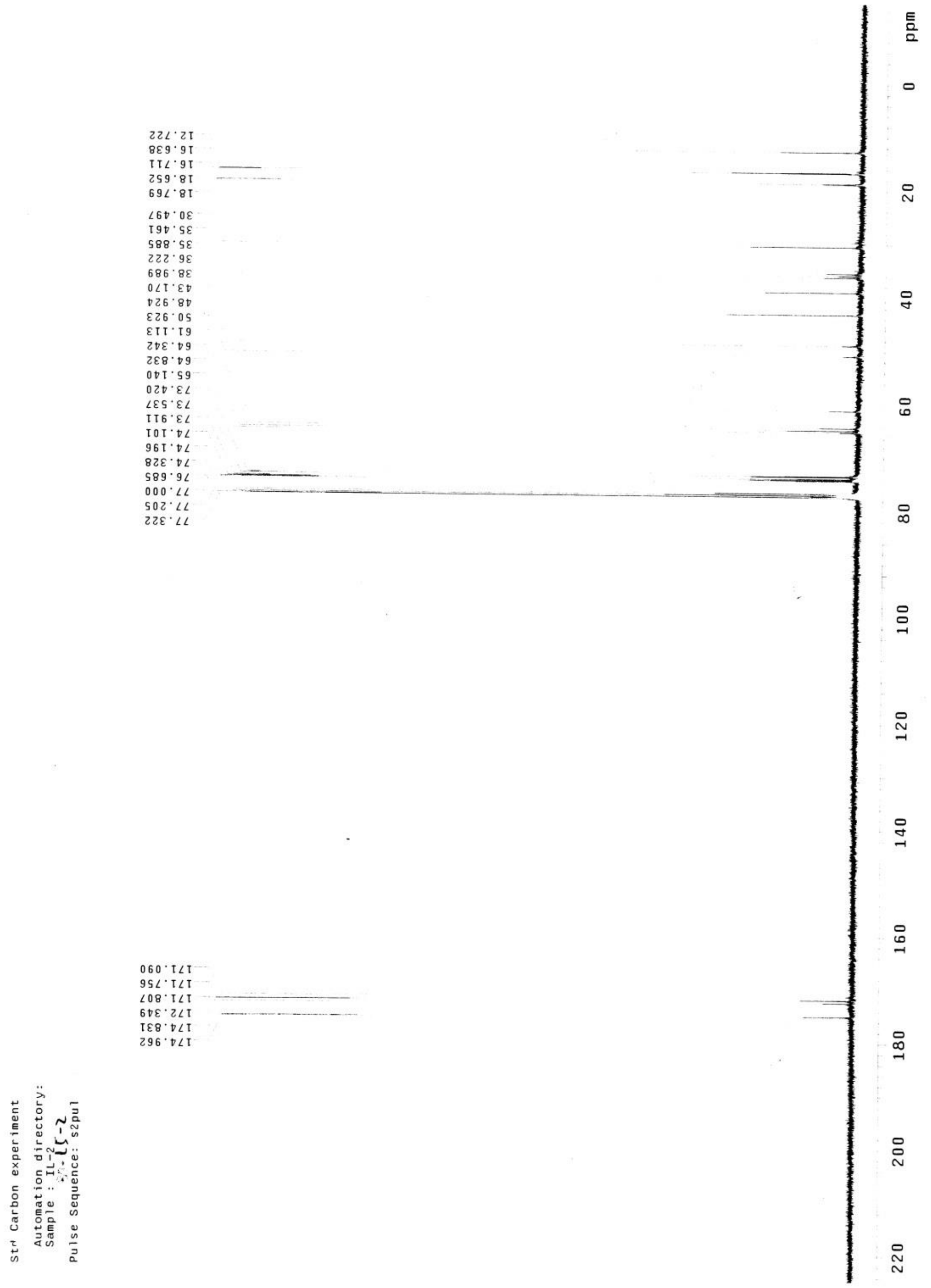

COSY spectrum of Gonocarin C （3）

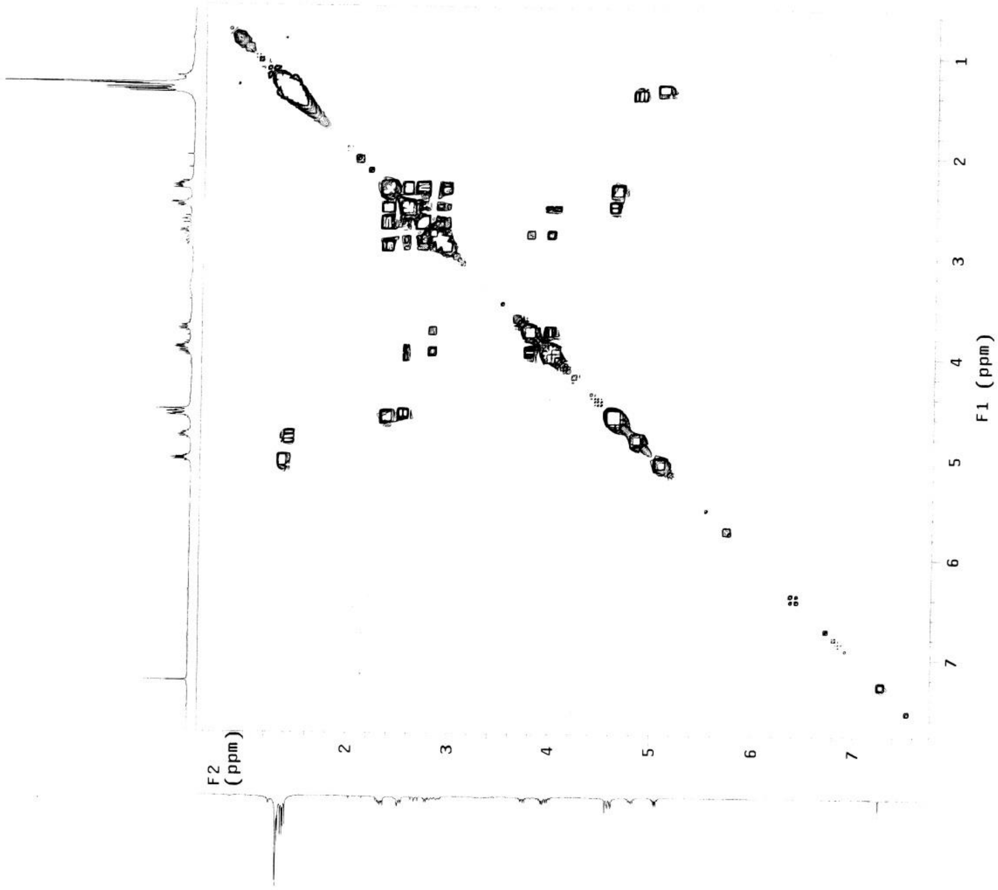

Std Proton parameters

Automation directory:
Sample : wei-E1-9-440-470-E2-H IL-2

Pulse Sequence: gCOSY

Solvent: CDCl3
Temp. 25.0 C / 298.1 K
Operator: sung
Mercury-400BB "NMMBA"

Relax. delay 1.000 sec
Acq. time 0.160 sec
Width 6406.1 Hz
2D Width 6406.1 Hz
32 repetitions
128 increments
OBSERVE H1, 400.4300877 MHz
DATA PROCESSING
Sq. sine bell 0.080 sec
F1 DATA PROCESSING
Sq. sine bell 0.020 sec
FT size 2048 x 2048
Total time 1 hr, 28 min, 48 sec

HMQC spectrum of Gonocarin C （3）

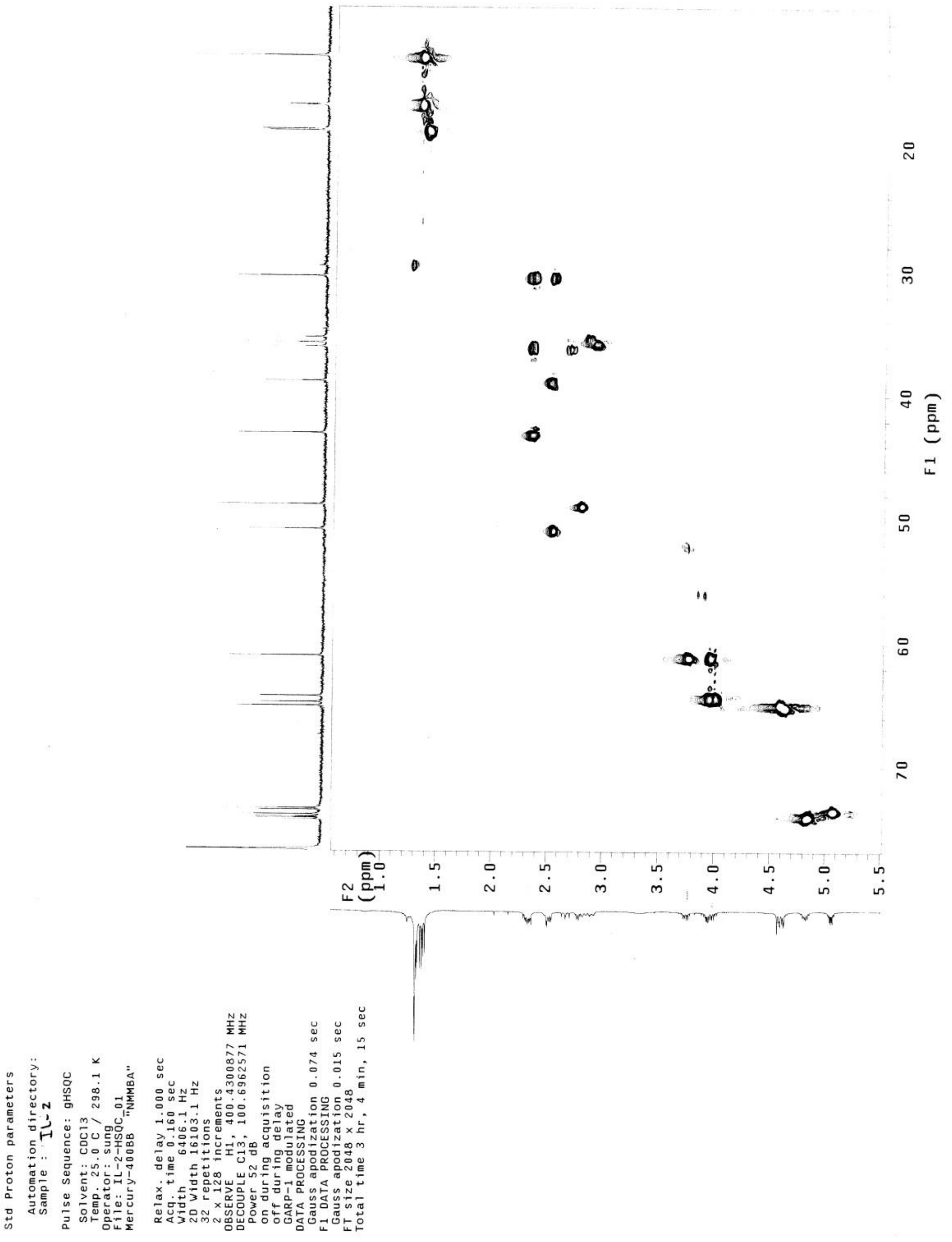

HMBC spectrum of Gonocarin C (3)

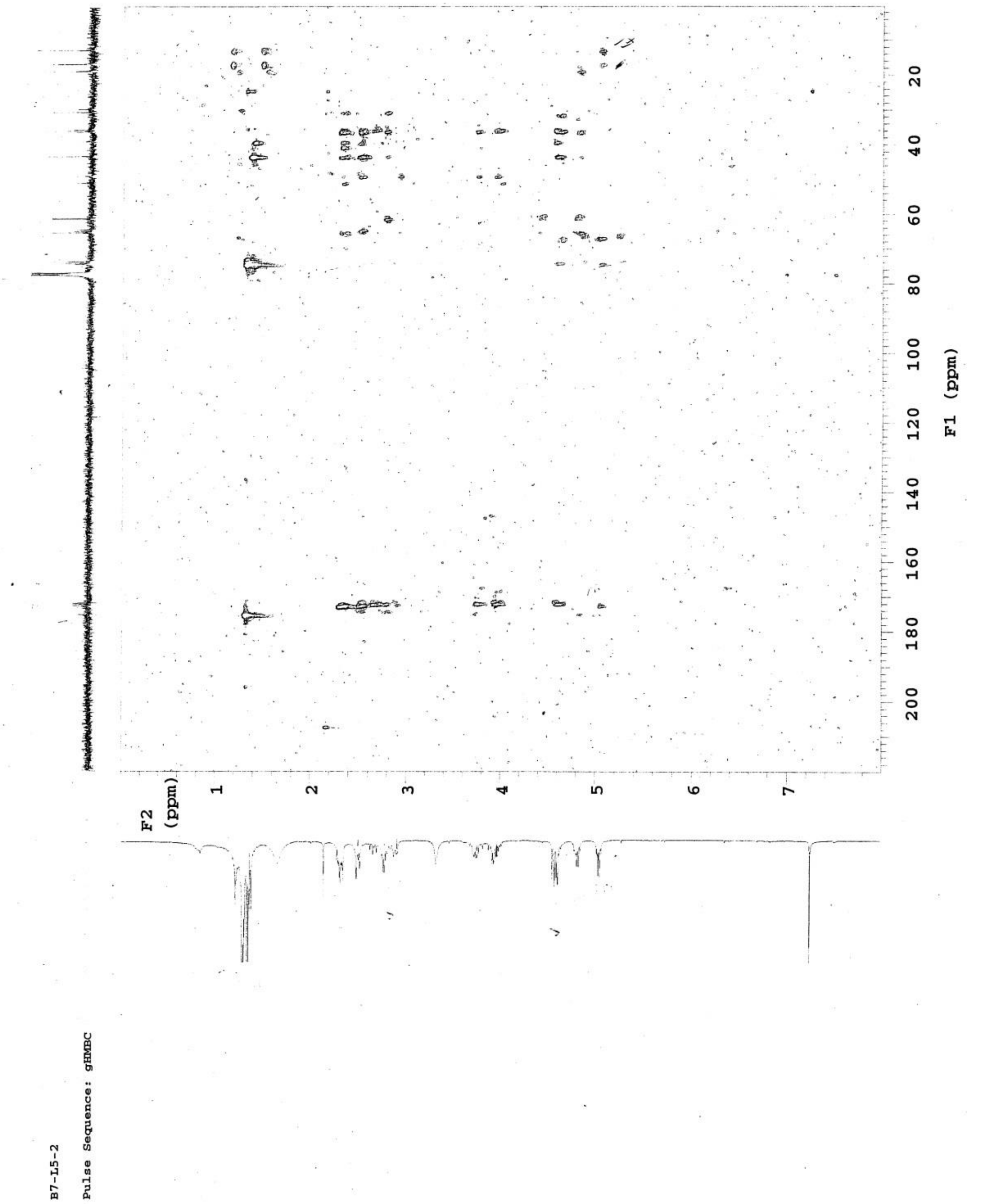

NOESY spectrum of Gonocarin C （3）

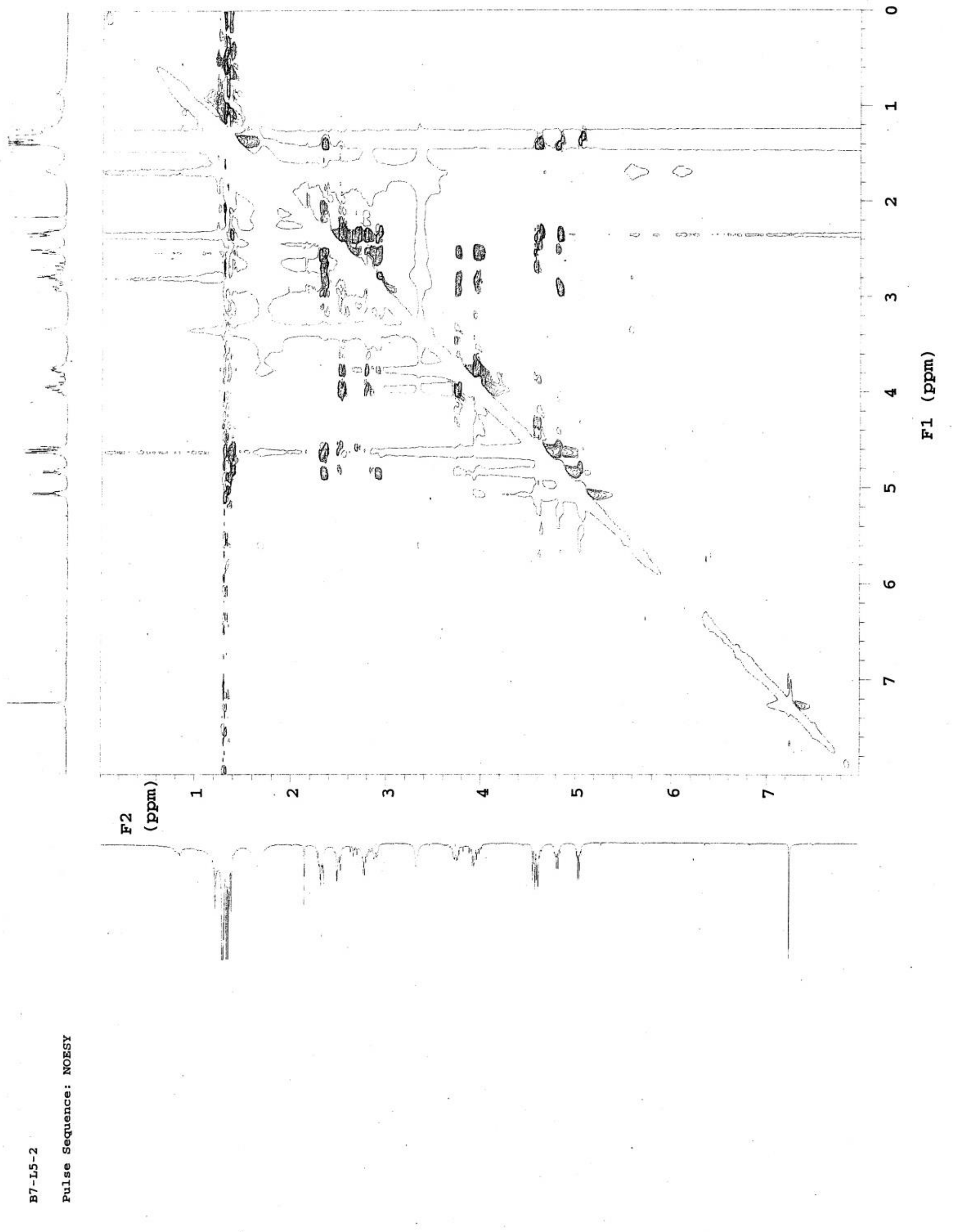

IR spectrum of Gonocarin C （3）

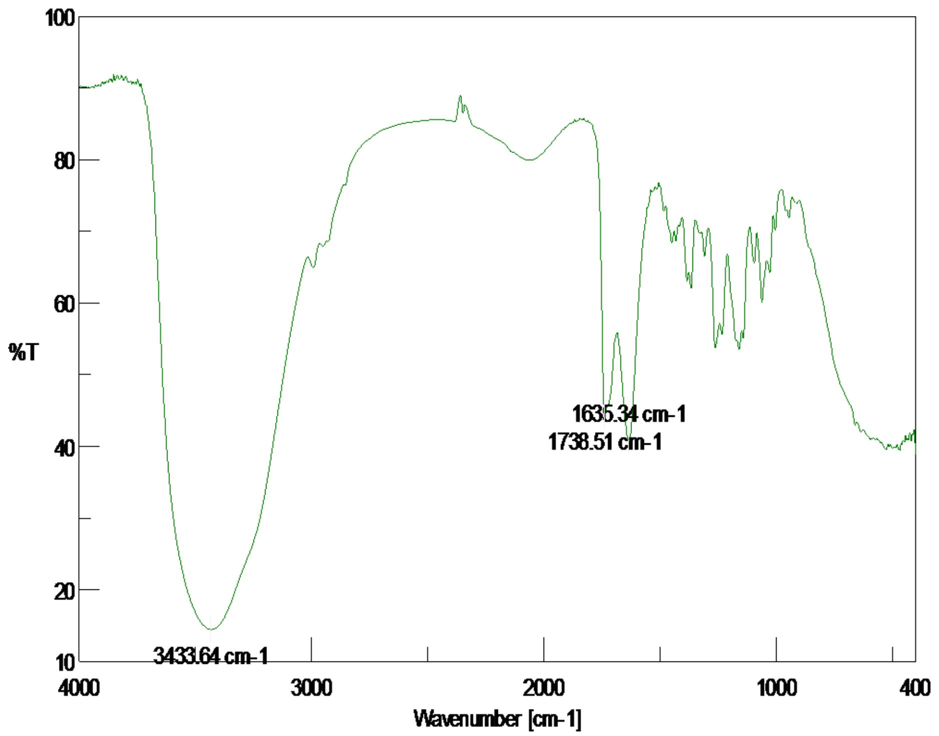

HR-ESI-MS spectrum of Gonocarin C （3）

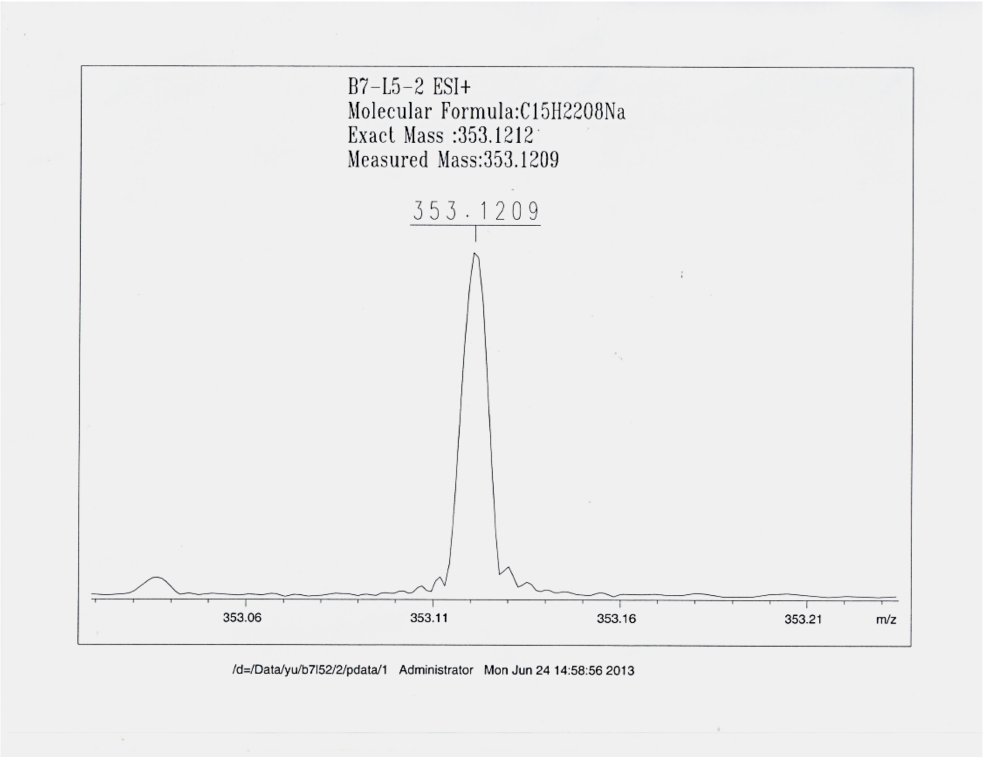

^{1}H NMR spectrum of Gonocarin A monoacetate （4）

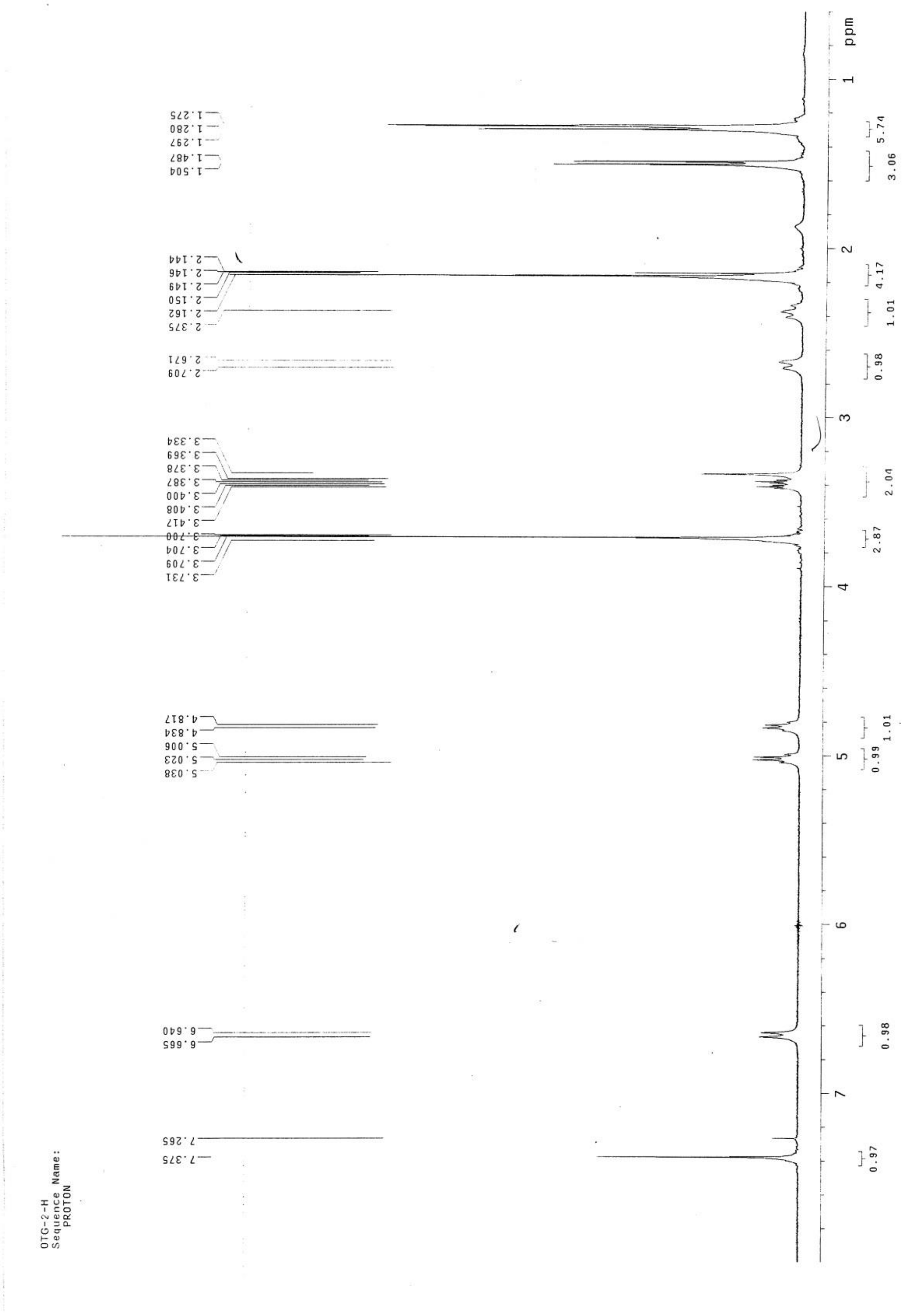

^{13}C NMR spectrum of Gonocarin A monoacetate （4）

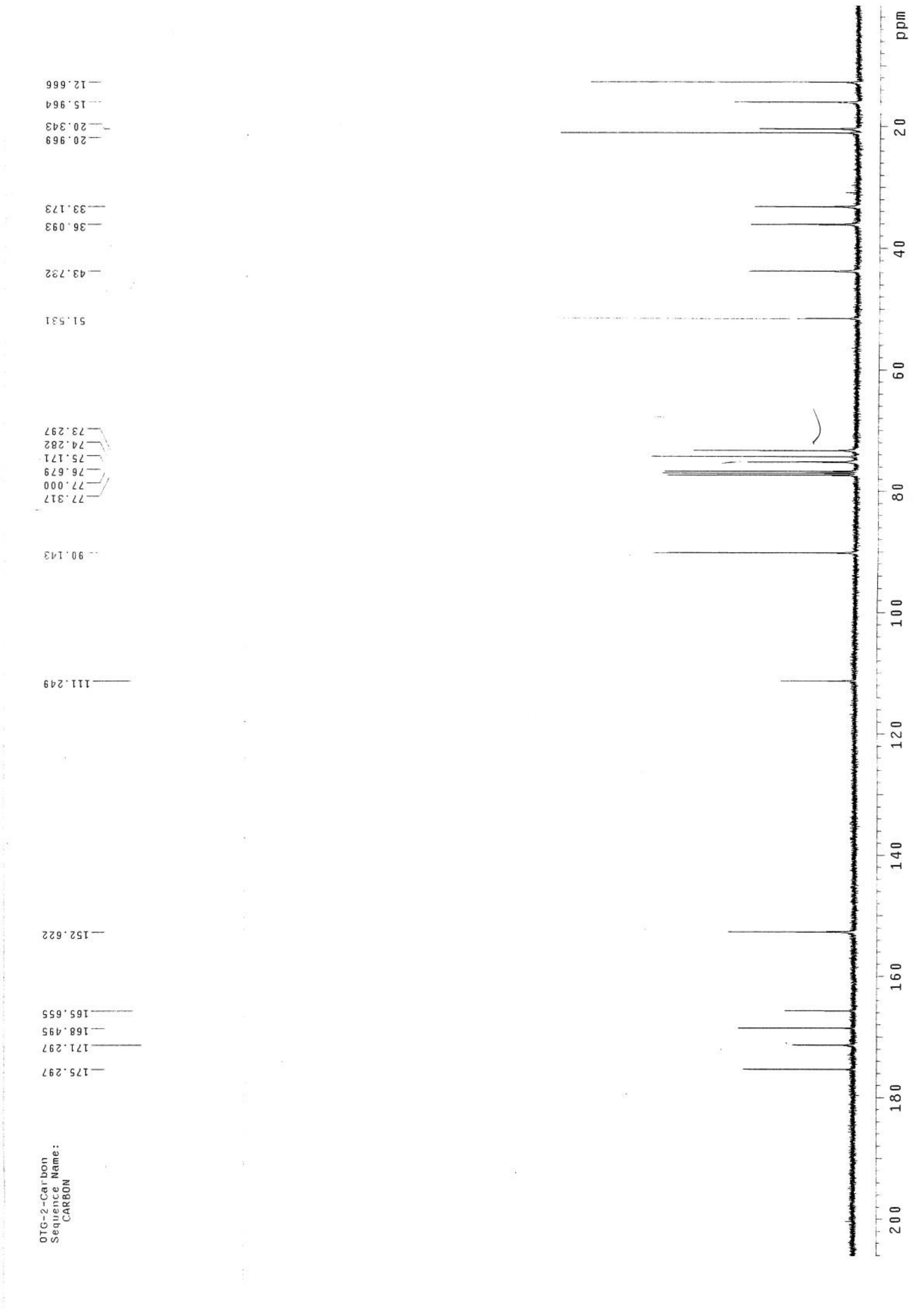

COSY spectrum of Gonocarin A monoacetate （4）

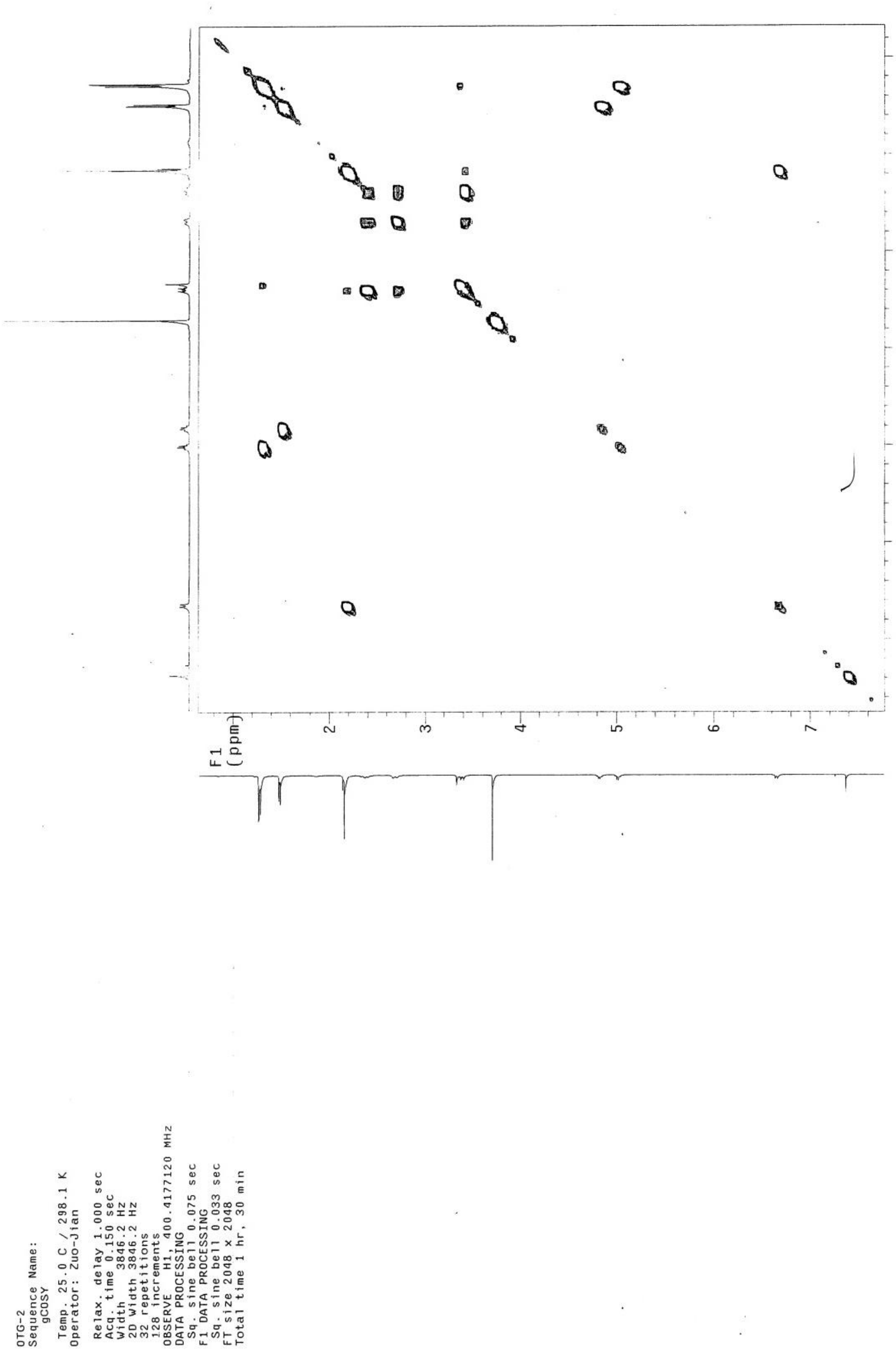

HMQC spectrum of Gonocarin A monoacetate （4）

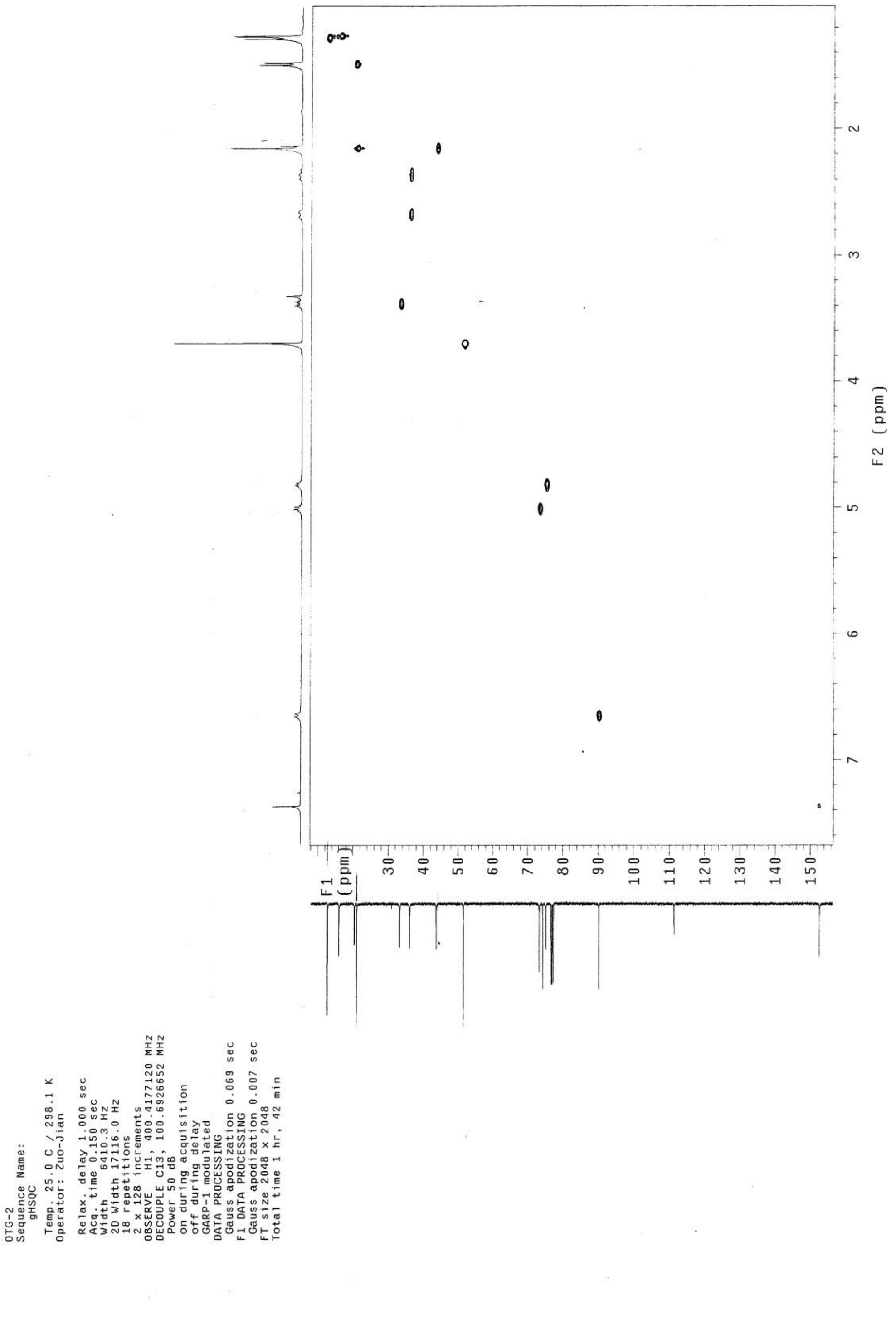

HMBC spectrum of Gonocarin A monoacetate （4）

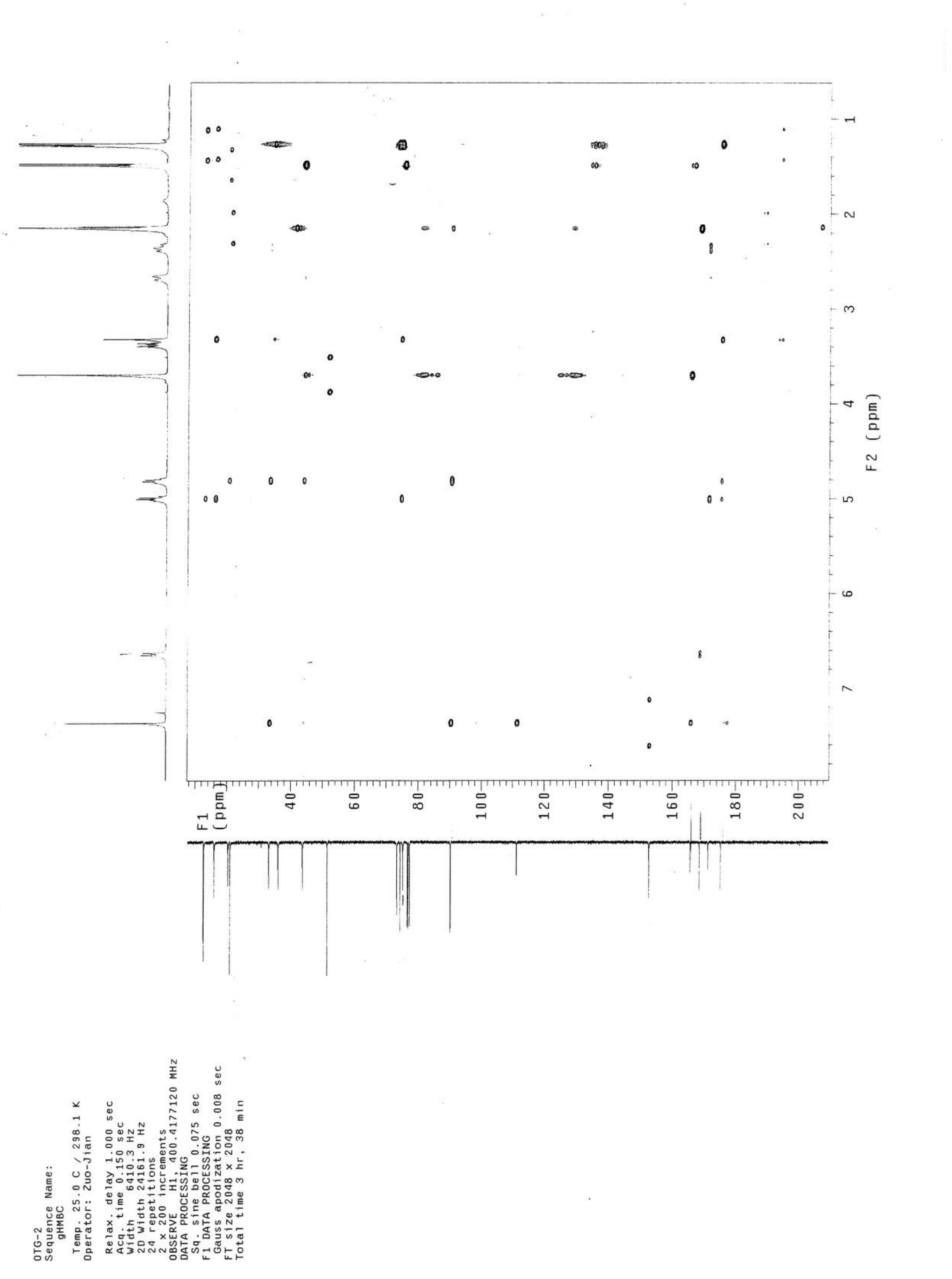

NOESY spectrum of Gonocarin A monoacetate （4）

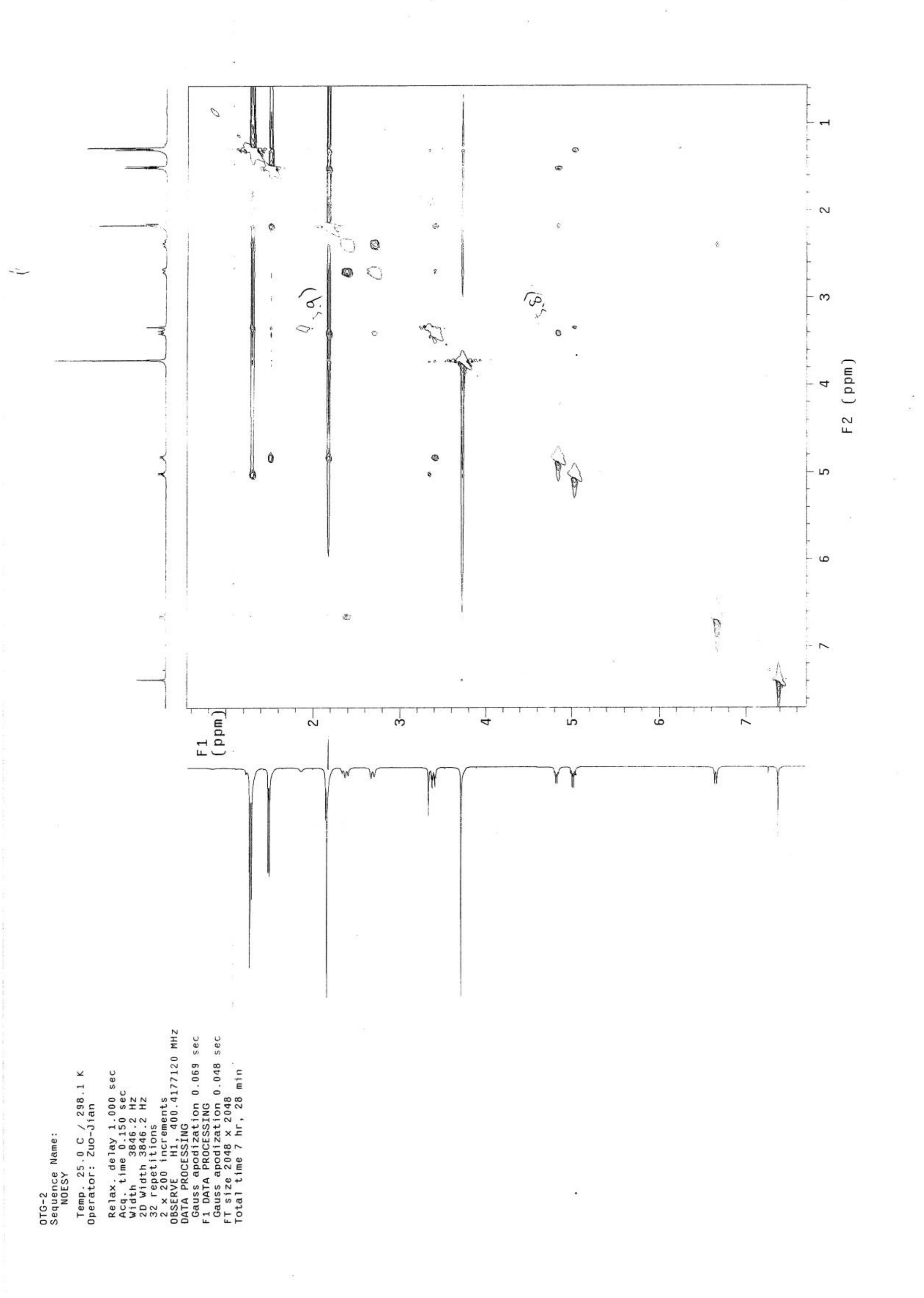

IR spectrum of Gonocarin A monoacetate (4)

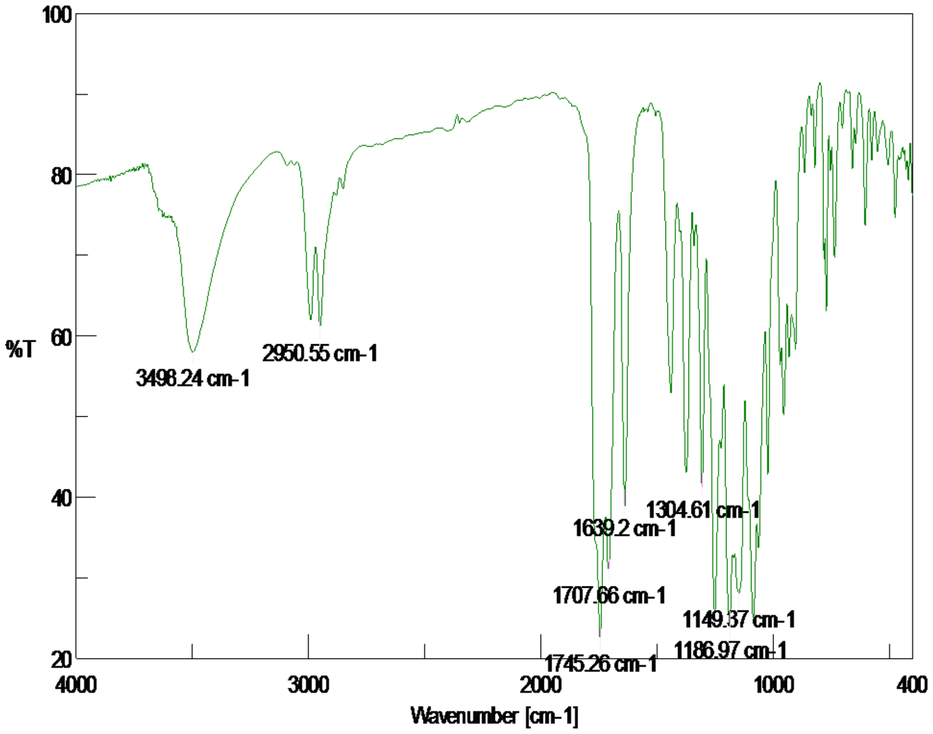

HRESIMS spectrum of Gonocarin A monoacetate (4)

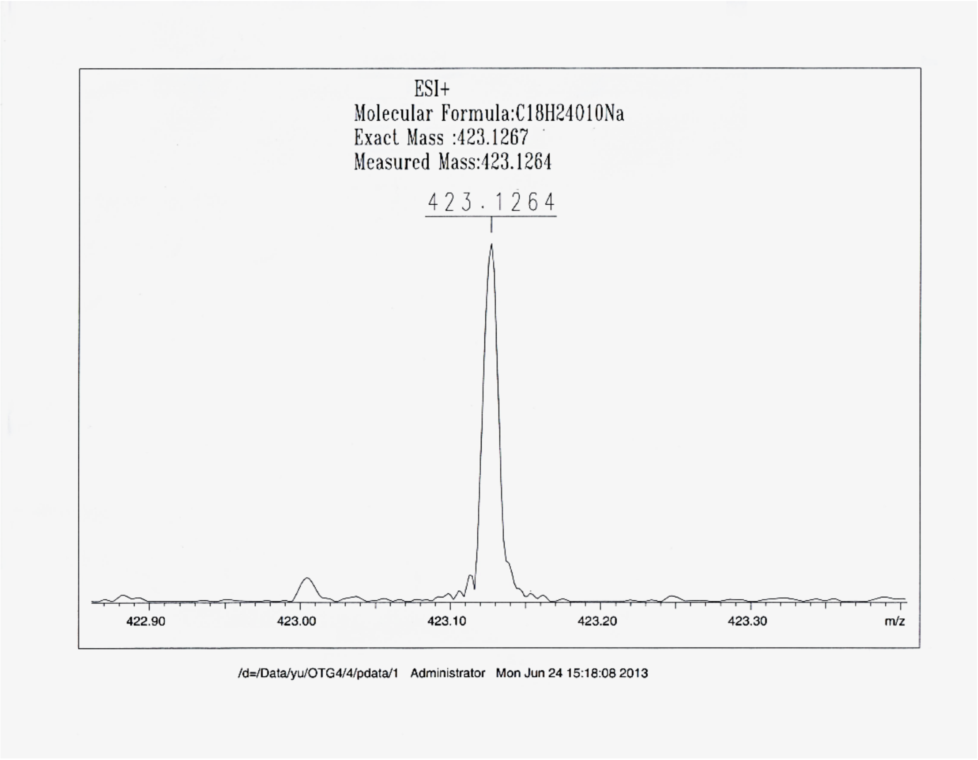

^{1}H NMR spectrum of Pinoresinol （5）

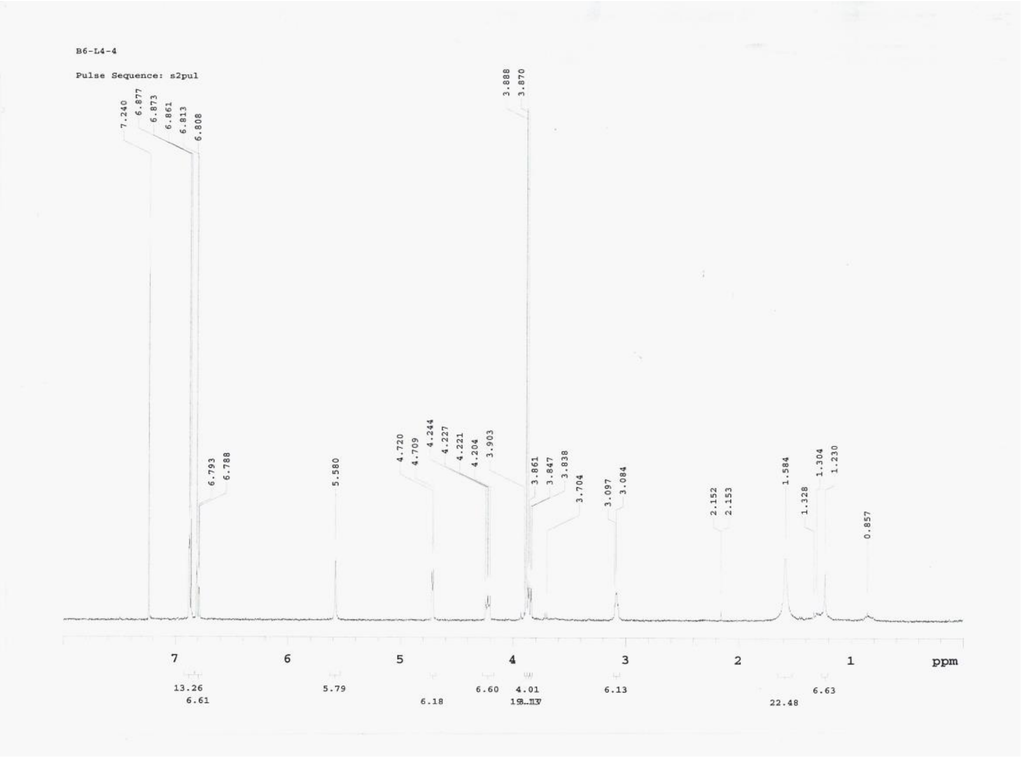

^{13}C NMR spectrum of Pinoresinol （5）

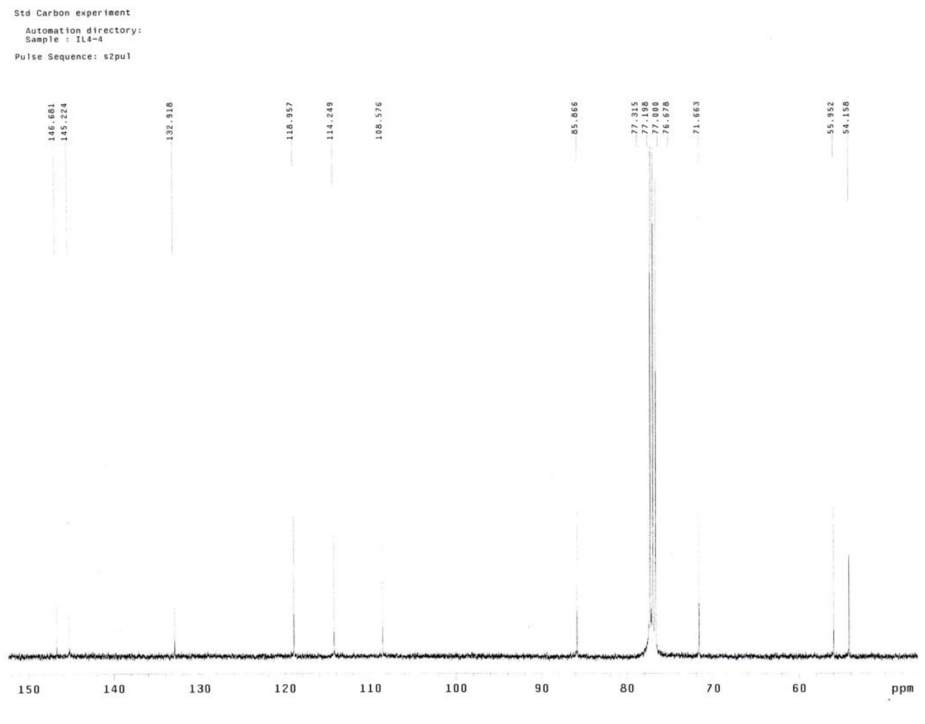

^{1}H NMR spectrum of Paulownin （6）

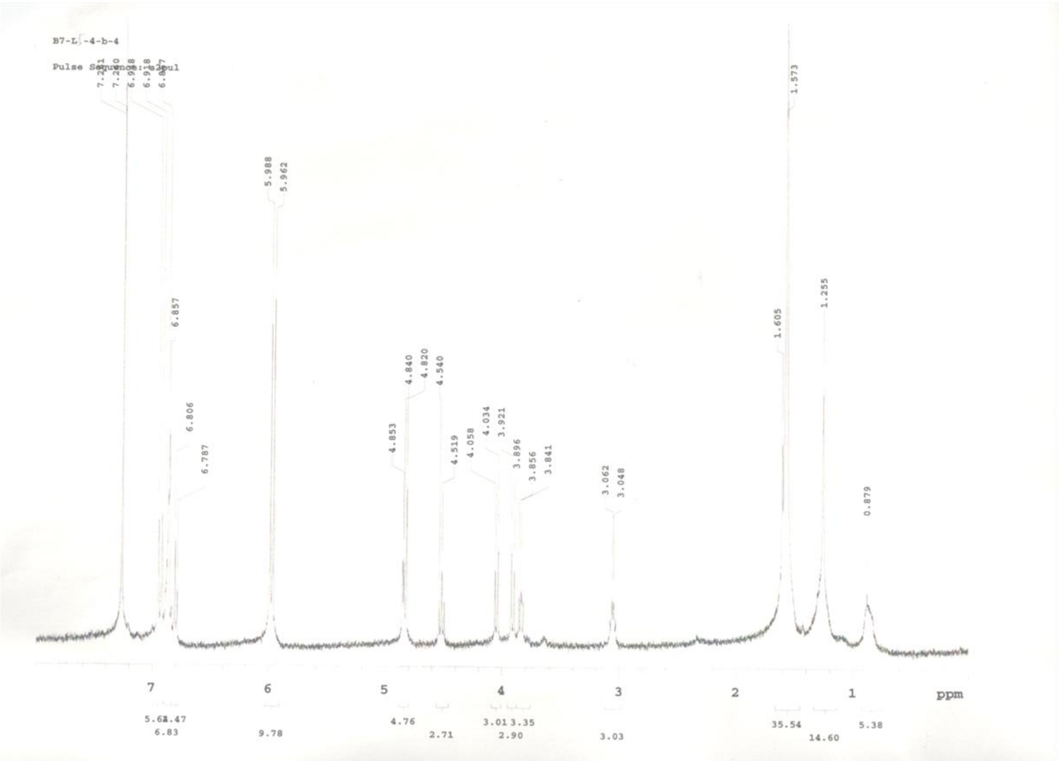

^{13}C NMR spectrum of Paulownin （6）

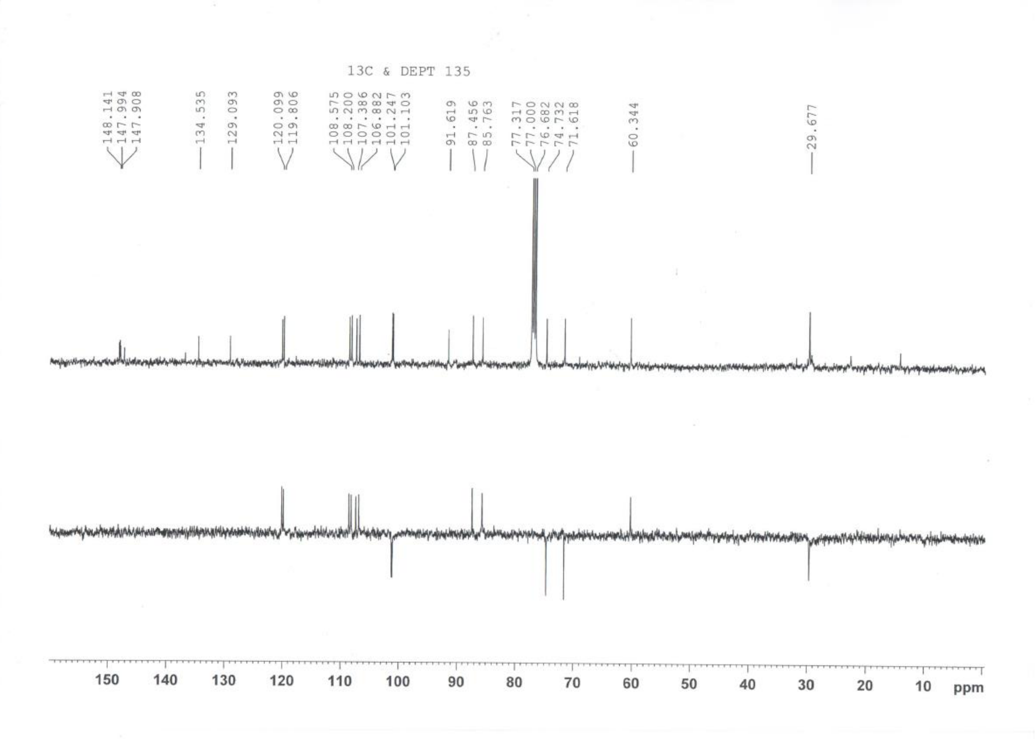

A

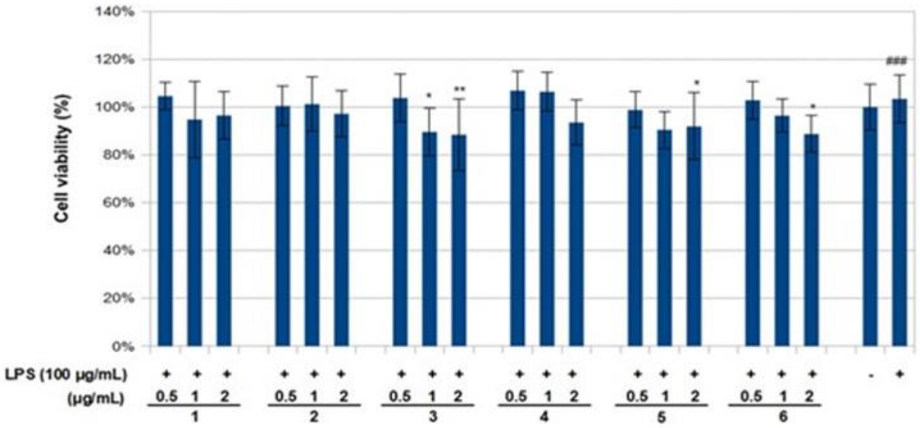

Figure 4. Effects of compounds **1-6** and LPS on the viability of RAW 264.7 macrophages. Cells were treated with LPS for 6 h prior to treatment with indicated concentrations of compounds or LPS alone. Following a 24h incubation period, cell viability was assessed using a MTS assay. Cell viability was calculated as the percentage of surviving cells over control cells (no compounds added). Values are presented as mean ± standard deviation of three independent experiments. ### p < 0.001 control group as compared to LPS-treated group. *p < 0.05, **p < 0.01, and ***p < 0.001 were compared with the LPS-alone group. - : cells without treatment, + : cells previously treated with LPS.

B

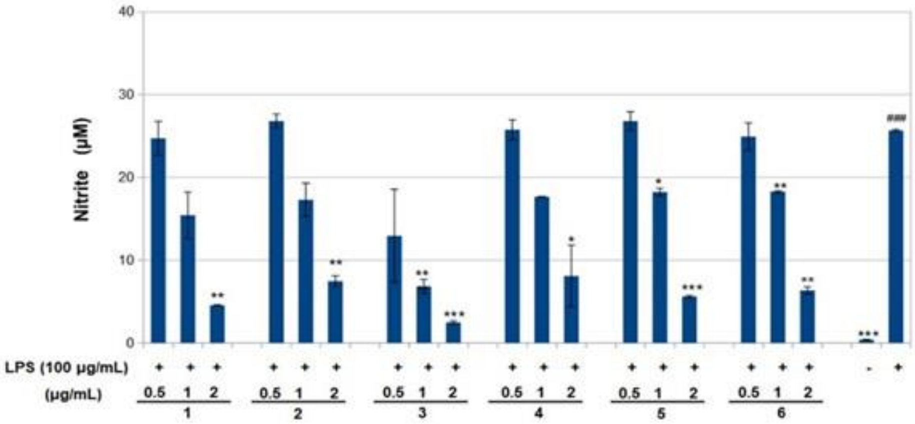

Figure 5. Inhibition of NO production by compounds **1-6** in LPS-stimulated RAW 264.7 macrophages. RAW 264.7 cells were stimulated by LPS (100 ng/ml) for 6h and then tetreated with the indicated concentrations of compounds 1-6 for 24 h. NO was measured using Griess reagent. Values are presented as mean ± standard deviation of three independent experiments. ### p < 0.001 control group as compared to LPS-treated group. *p < 0.05, **p < 0.01, and ***p < 0.001 were compared with the LPS-alone group. - : cells without treatment, + : cells previously treated with LPS.

C

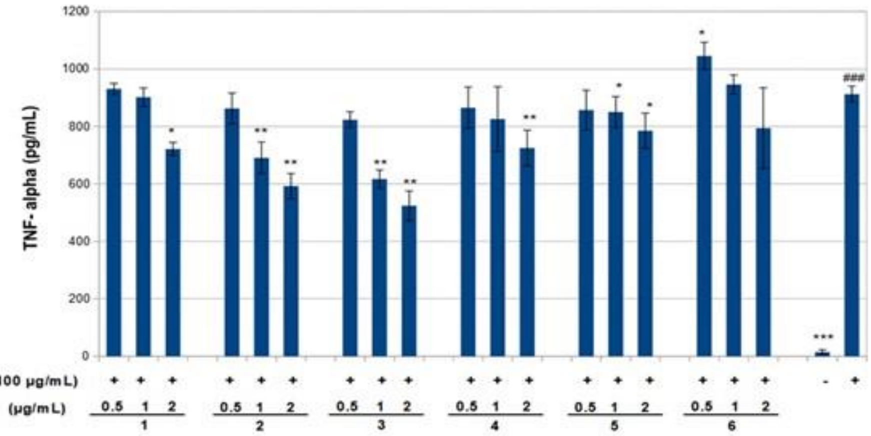

Figure 6. Inhibition of TNF-α production by compounds **1**-**6** in LPS-stimulated RAW 264.7 macrophages. RAW 264.7 cells were stimulated by LPS (100 ng/ml) for 6 h then treated with various concentrations of compounds 1-6 for 24 h. TNF-α production were measured using the corresponding ELISA kits. Values are presented as mean ± standard deviation of three independent experiments. ### $p < 0.001$ control group as compared to LPS-treated group. *$p < 0.05$, **$p < 0.01$, and ***$p < 0.001$ were compared with the LPS-alone group. - : cells without treatment, + : cells previously treated with LPS.
